# Surviving Endoplasmic Reticulum Stress Is Coupled to Altered Chondrocyte Differentiation and Function 

**DOI:** 10.1371/journal.pbio.0050044

**Published:** 2007-02-13

**Authors:** Kwok Yeung Tsang, Danny Chan, Deborah Cheslett, Wilson C. W Chan, Chi Leong So, Ian G Melhado, Tori W. Y Chan, Kin Ming Kwan, Ernst B Hunziker, Yoshihiko Yamada, John F Bateman, Kenneth M. C Cheung, Kathryn S. E Cheah

**Affiliations:** 1 Department of Biochemistry, University of Hong Kong, Pokfulam, Hong Kong, China; 2 Department of Orthopaedics and Traumatology, University of Hong Kong, Pokfulam, Hong Kong, China; 3 ITI Research Institute for Dental and Skeletal Biology, University of Bern, Bern, Switzerland; 4 Craniofacial Developmental Biology and Regeneration Branch, National Institute of Dental and Craniofacial Research, Bethesda, Maryland, United States of America; 5 Murdoch Childrens Research Institute, Royal Children's Hospital, Melbourne, Victoria, Australia; 6 Department of Paediatrics, University of Melbourne, Melbourne, Victoria, Australia; California Institute of Technology, United States of America

## Abstract

In protein folding and secretion disorders, activation of endoplasmic reticulum (ER) stress signaling (ERSS) protects cells, alleviating stress that would otherwise trigger apoptosis. Whether the stress-surviving cells resume normal function is not known. We studied the in vivo impact of ER stress in terminally differentiating hypertrophic chondrocytes (HCs) during endochondral bone formation. In transgenic mice expressing mutant collagen X as a consequence of a 13-base pair deletion in *Col10a1 (13del),* misfolded α1(X) chains accumulate in HCs and elicit ERSS. Histological and gene expression analyses showed that these chondrocytes survived ER stress, but terminal differentiation is interrupted, and endochondral bone formation is delayed, producing a chondrodysplasia phenotype. This altered differentiation involves cell-cycle re-entry, the re-expression of genes characteristic of a prehypertrophic-like state, and is cell-autonomous. Concomitantly, expression of *Col10a1* and *13del* mRNAs are reduced, and ER stress is alleviated. ERSS, abnormal chondrocyte differentiation, and altered growth plate architecture also occur in mice expressing mutant collagen II and aggrecan. Alteration of the differentiation program in chondrocytes expressing unfolded or misfolded proteins may be part of an adaptive response that facilitates survival and recovery from the ensuing ER stress. However, the altered differentiation disrupts the highly coordinated events of endochondral ossification culminating in chondrodysplasia.

## Author Summary

The assembly and folding of secreted proteins in the endoplasmic reticulum (ER) is exquisitely regulated by a complex mechanism that maintains an equilibrium between folded and unfolded proteins. Perturbation of this homeostasis induces ER stress, which, if not alleviated through ER stress signaling (ERSS), ultimately triggers cell death. Normal bone growth occurs through a highly coordinated differentiation program that yields specialized cartilage cells (chondrocytes); when this program is disrupted, chondrodysplasia, or malformed skeletons, can result. Chondrodysplasias caused by mutations that affect protein assembly and secretion are characterized by a disorganization of bony growth plates and distension of the ER. We tested whether these chondrodysplasia characteristics were linked to ERSS. By investigating the impact of ER stress on the cell fate of hypertrophic chondrocytes (HCs) in transgenic mice expressing mutations in collagen that prevent proper folding, we revealed a novel adaptive mechanism that helps alleviate the unfolded protein load. Instead of undergoing apoptosis, the HCs undergoing ER stress adapt, re-enter the cell cycle, and revert to a less-mature state in which expression of the mutant collagen is reduced. Our findings have broad implications for adaptive mechanisms to ER stress in vivo and for the pathophysiology underlying chondrodysplasias caused by mutations that impact on protein assembly and secretion.

## Abstract 3

When subjected to ER stress (by expression of misfolded or unfolded proteins), hypertrophic chondrocytes undergo alterations to their developmental program that may be part of an adaptive response.

## Introduction

Development and growth require the ability to detect, respond to, and survive stresses that compromise the normal state. Accumulation of misfolded or unfolded mutant proteins in the endoplasmic reticulum (ER) induces ER stress and may seriously affect the viability of cells. To cope with ER stress, ER-resident sensors detect misfolded or unfolded proteins and elicit the ER stress signaling (ERSS), which includes the induction of the highly conserved “unfolded protein response” (UPR). ERSS may lead to cytoprotection or death, depending on the nature of the stress and the cellular context.

ERSS involves the activation of at least three independent ER stress sensors: inositol-requiring 1 (IRE1), PKR-like ER kinase (PERK), and membrane-tethered activating transcription factor 6 (ATF6) [1]. Their activation represses protein synthesis via phosphorylation of the translation initiation factor eIF2α and activates signaling pathways that up-regulate the expression of ER-resident molecular chaperones and translation regulators. Activation of IRE1, PERK, and ATF6 depends on their dissociation from the molecular chaperone, binding Ig protein (BiP), a master regulator of ERSS. BiP ensures high-fidelity protein folding and prevents the accumulation of unfolded or aggregated proteins. Upon stress, unfolded proteins bind BiP and sequester it from interaction with IRE1, PERK, and ATF6. The released ATF6 and IRE1 activate the transcription factor XBP1 via production of its spliced form, XBP1^s^ [2,3]. In an auto-regulatory loop, BiP activity increases further, since BiP up-regulation is partially dependent on XBP1^s^ [4]. Induction of ERSS means that the amount of new protein translocated into the ER lumen is reduced, degradation of ER-localized proteins increases, and protein-folding capacity is enhanced.

ERSS is triggered in a range of pathogenic conditions, e.g., nutrient deprivation, viral infection, and expression of mutant secretory or membrane-bound proteins, that saturate the folding machinery, leading to overload of the ER (reviewed in [1]). It has been suggested that physiological ER load and ERSS components regulate cellular differentiation and developmental decisions: e.g., terminal plasma cell differentiation (reviewed in [5]) and bone and cartilage development [6]. ER stress has been implicated in many diseases, such as neurodegenerative disorders (Pelizaeus-Merzbacher disease [7] and amyotrophic lateral sclerosis [8]), osteogenesis imperfecta [9], and leukemia [10].

Despite the detailed description of the molecular pathways in ERSS, many questions remain regarding its impact in vivo. If ER stress is not alleviated, an apoptotic program is initiated [11], but how cells alleviate ER stress in vivo is not well understood. We addressed these questions by investigating the in vivo impact of ER stress on a well-defined and clinically important developmental pathway, the terminal differentiation of chondrocytes during endochondral bone formation.

This depends on a highly coordinated program of differentiation, proliferation, maturation by permanent withdrawal from the cell cycle, hypertrophy, and terminal differentiation of chondrocytes within the mammalian growth plate. A snapshot of this differentiation program is captured in histological sections taken through the developing growth plate in which the differentiation stages are reflected by morphologically distinct subpopulations of chondrocytes organized in a spatial and columnar pattern and in defined relative proportions (reviewed in [12,13]). Recognizable subpopulations of chondrocytes are round resting/reserve cells; flattened proliferating cells organized into columns; larger, non-dividing (mature) prehypertrophic cells (preHCs), and the even larger terminally differentiated hypertrophic chondrocytes (HCs), which undergo apoptosis as the cartilage matrix mineralizes and is being replaced with bone. This program is regulated by a complex network of molecules such as Indian hedgehog (IHH), parathyroid hormone-related peptide (PTHrP), bone morphogenetic proteins, fibroblast growth factors (FGFs), their respective receptors, and interaction between the cells and the extracellular matrix (ECM) [12,13].

Perturbation of this differentiation program results in a malformed skeleton (chondrodysplasia). Distension of the ER is a hallmark of cells undergoing ER stress [14,15]. Disorganization of growth plate chondrocytes and distended ER are characteristics of several chondrodysplasias [16–18], many of which are caused by mutations in genes encoding ECM proteins, such as collagens II, IX, XI, cartilage oligomeric matrix protein (COMP), and aggrecan (reviewed in [19]) and that result in the synthesis of abnormal proteins. Cultured pseudochondroplastic chondrocytes or cells transfected with mutant COMP retain ECM proteins and chaperones intracellularly and have an increased capacity for apoptosis [14,16,20]. Collagen X is an ECM protein synthesized specifically by HCs. Mutations in the human *COL10A1* gene interfere with collagen X trimer assembly and secretion, with retention of mutant chains in the ER [21–23], resulting in metaphyseal chondrodysplasia type Schmid (MCDS) (reviewed in [24,25]). Abnormal organization of chondrocytes and an expanded hypertrophic zone (HZ) have been observed in the growth plate of a swine model for MCDS [26].

These observations raise the possibility that in chondrodysplasias caused by mutations that affect protein assembly and secretion, ERSS disrupts chondrocyte differentiation and thereby chondrocyte organization in the growth plate. In this study, we present evidence that when unfolded collagen X encoded by a murine *Col10a1*-equivalent of an MCDS mutation, a 13-base pair (bp) deletion within the NC1-encoding domain [27], is expressed in transgenic mice (13del), ERSS is triggered in the growth plate. Concomitantly, HC fate and terminal differentiation are perturbed, but apoptosis is not increased. The normal terminal differentiation program is disrupted. The 13del HCs in the lower part of the HZ re-enter the cell cycle and express markers more typical of preHCs, accompanied by down-regulation of *Col10a1-13del* gene (hereafter referred to as *13del*) expression, suggesting an alteration in their cellular identity. This alteration of 13del HCs is cell autonomous, implying an association with activation of the ERSS. These findings reveal for the first time that HCs undergoing ER stress in vivo adapt, altering their differentiation status to a less-mature state in which expression of *13del* is reduced, thereby alleviating the unfolded protein load.

## Results

### The *Col10a1* 13del Mutation Results in Impaired Collagen X Assembly

**Figure 1:**
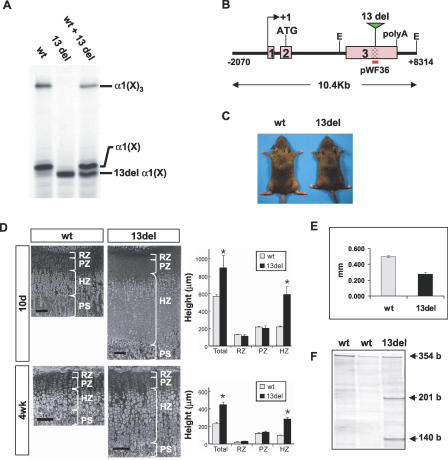
Impaired Collagen X Assembly and Growth Plate Abnormality (A) Cell-free translation demonstrating normal assembly of wt α-chains into α1(X)_3_ homotrimers, but impaired assembly of 13del α-chains into trimers. Heterotrimers, which would migrate slightly faster than the α1(X)_3_ homotrimers, are absent. (B) Diagram of the 10.4-kb *Col10a1-13del* transgene. The NC1-encoding domain is indicated by the shaded region. (C) Ten-week-old wt and 13del mice. (D) Left: histology of the proximal tibial growth plates of 10-d-old (10d) and 4-wk-old (4wk) wt and 13del mice. Right: differences in the total height of the growth plate and in the heights of the RZ, PZ, and HZ between wt and 13del mice in each age group (10 d [*n* = 6] and 4 wk [*n* = 13]). Bar indicates 100 μm. (E) Retarded longitudinal bone growth in 13del mice. Calcein (10 mg/kg body weight) was injected intraperitoneally into 5-d-old mice that were sacrificed 5 d later. Sections of proximal tibia were examined in an incident-light fluorescence microscope. The distances at different positions between the zone of vascular invasion at the growth plate and the proximal endpoint of the calcein label in the trabecular bone were determined using a micrometric eyepiece. The mean distance represented the rate of longitudinal bone growth over 5 d. Error bars represent ±1 standard deviation (SD). Longitudinal growth was retarded in 13del mice over 5–10 d, being only about half that in wt littermates (0.498 ± 0.013 mm for wt and 0.277 ± 0.025 mm for 13del; *n* = 3 for each genotype; *p* = 5.2 × 10^−6^). (F) RNase protection assay. The protected fragments of [α-^33^P]UTP-labeled probes when hybridized with wt *Col10a1* transcript are 354 bases; whereas hybridization with 13del transcripts gives two fragments of 201 bases and 140 bases (the unpaired 13 bases are digested by RNase). PS, primary spongiosa.

Trimer assembly of collagen X α-chains via the NC1 domain is critical for triple helix formation and for secretion [24]. We tested the effect of the 13del mutation on collagen X trimer assembly in vitro by cell-free translation of wild-type (wt) and 13del mRNAs. Consistent with previous findings [21,22], the mutation resulted in collagen X α-chains that were unable to assemble into trimers (Figure 1A). Co-translation of wt *Col10a1* and 13del mRNAs resulted in fewer collagen X trimers, suggesting 13del α-chains reduced trimer assembly (Figure 1A). Thus, this mutation would be expected to impair collagen X folding/assembly in vivo.

### Abnormal Chondrocyte Differentiation in 13del Transgenic Mice

To assess the impact of unfolded collagen X α-chains on the differentiation of the chondrocyte layers in vivo, we generated transgenic mice carrying the *13del* (Figure 1B). Five independent lines carrying the *13del* transgene all exhibited disproportionate dwarfism with shorter limbs (Figure 1C). Histologic and morphometric analyses revealed altered proportional organization of the growth plate with a significant expansion specifically of the HZ (Figure 1D). This expansion was detectable in 13del growth plates at late fetal stage, peaking on about day 10 (*p* = 0.015, *n* = 6), and diminishing by 4 wk (*p* < 0.001, *n* = 13). By 10 wk, the height was normal and corresponded to the cessation of transgene expression (see below). Longitudinal bone growth as measured by calcein labeling was significantly reduced in 13del mice at 5- to 10-d-old (Figure 1E). At 10 wk, 13del tibiae were 15% shorter than that of the wt (*n* = 12, *p* = 4 × 10^−14^). These results indicated that the phenotypic changes were associated with disturbed endochondral ossification.

**Figure 2:**
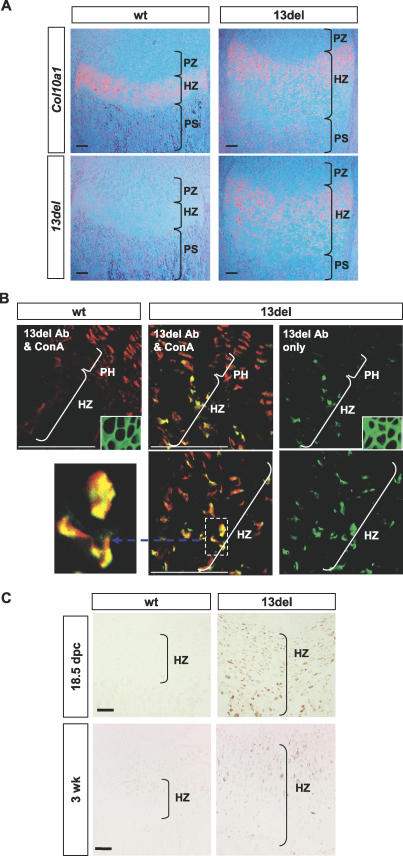
Intracellular Accumulation of 13del Proteins (A) In situ hybridization for wt *Col10a1* or *13del* transcripts at 10-d-old proximal tibial growth plates. (B) Immunofluorescence of cryosections immunostained with a 13del-specific antibody (13del Ab, green fluorescence). Absence of staining in the HZ of wt littermates showed the antibody is specific for 13del protein (Figure 1G, wt panel). Red fluorescence signal marks concanavalin A (ConA) in the ER. Localization of 13del proteins within the ER (yellow) is shown by the overlapping green and red fluorescence signals. Insets show extracellular immunostaining for normal collagen X in wt and 13del mice. (C) Immunohistochemistry using 13del-specific antibody on paraffin-embedded proximal tibial growth plate sections. Specific staining (brown) was seen only in 13del HZ. The sections were not counterstained in order to access the intensity of staining more easily. Bar indicates 100 μm.

Transgenic mice overexpressing wt *Col10a1* in HCs under the identical regulatory sequences are phenotypically normal (unpublished data). Hence, the observed expansion in the HZ is consistent with a defect induced by the expression of the *13del* transgene. RNase protection assays confirmed the expression of the *13del* transgene (Figure 1F). The expansion of the HZ was relatively greater in another 13del line showing higher transgene expression and was less in yet another line with lower *13del* expression (Figure S1), indicating a dosage-dependent effect. In situ hybridization using *Col10a1*- and *13del*-specific riboprobes revealed co-expression of *13del* mRNA with endogenous *Col10a1* specifically in the 13del HCs (Figure 2A). In wt littermates, *Col10a1* expression was largely restricted to, and maintained at a high level in the HZ (Figure 2A). By contrast, the expression of both *Col10a1* and the *13del* transgene in 13del mice was down-regulated toward the lower portion of the hypertrophic zone (LHZ) (Figure 2A), indicating an alteration in the differentiation program of HCs.

### 13del HCs Retain 13del Proteins Intracellularly and Survive ER Stress

Immunostaining with a 13del-specific antibody showed 13del proteins were restricted to the HCs in the growth plate. The 13del proteins were intracellular, with little or no staining in the ECM (Figure 2B, 13del panels), and co-localized with concanavalin A, which binds to carbohydrate moieties in the ER (Figure 2B, middle panels). Endogenous wt collagen X was secreted to the ECM in mutant as in wt mice (Figure 2B, insets). The 13del protein level varied both temporally and spatially: just before birth at 18.5 d post coitum (dpc), when the HZ expansion had not yet reached its maximum, mutant proteins appeared to be evenly present in the HZ (Figure 2C). By contrast, there was reduced amounts of mutant protein in the LHZ of 3-wk-old 13del mice (Figure 2C), a time point at which the degree of expansion of the HZ relative to wt was less than for 10-d-old mice.

**Figure 3:**
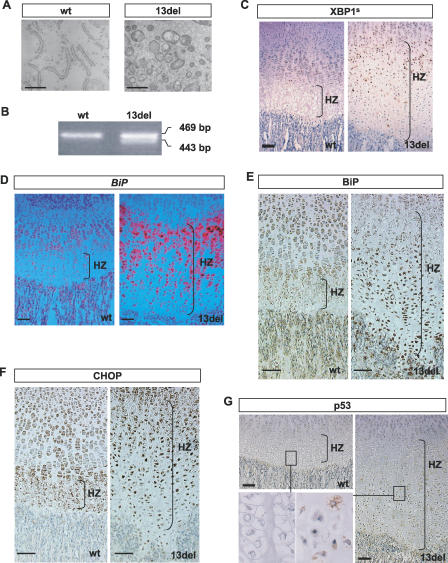
Induction of ERSS in 13del HCs Analyses of 10-d-old proximal tibial growth plates. (A) Electron micrographs of wt and 13del HCs showing distended and fragmented ER (bar indicates 1 μm). (B) RT-PCR of total RNA from hypertrophic cartilage: 469 bp: unspliced *Xbp1* mRNA; 443 bp: spliced *Xbp1^s^*. Sequencing of the 443-bp PCR-fragment confirmed that it is the product of *Xbp1^s^* mRNA (unpublished data). (C–E) Expression of XBP1^s^ and *BiP* revealed by in situ hybridization (D) and immunostaining (C and E). (F and G) Immunohistochemical detection of CHOP (F) and p53 (G). The boxes in (G) indicate the region of higher magnification shown in the bottom left corner. Color contrast of the low-power magnification images in (C–G) were adjusted as described in Materials and Methods. Bar indicates 100 μm.

Ultrastructural analysis revealed distended and fragmented ER in 13del HCs, but not in other growth plate zones (Figure 3A), raising the possibility that they were experiencing ER stress. We tested for expression of alternatively spliced *Xbp1* mRNA *(Xbp1^s^),* a major transducer of the ER stress signal. Both the spliced and unspliced isoforms of *Xbp1* were expressed in growth plate cartilage mRNA from 13del mice (Figure 3B), whereas only the unspliced form was found in wt littermates. Immunostaining revealed a strong induction of XBP1^s^ protein in HCs expressing 13del protein in 13del upper HZ (UHZ) compared with wt littermates (Figure 3C, Figure S3A). Expression of *Edem,* a direct downstream target gene of XBP1^s^ [28,29], was also induced in 13del HZ, but not in the wt littermates (Figure S2).

The mRNA for the molecular chaperone BiP is strongly expressed in the UHZ in 13del mice (Figure 3D), concomitant with the expression of the *13del* transgene (Figure 2A), and gradually less so in cells toward the LHZ. This is consistent with the higher level of unfolded proteins and induction of ERSS in 13del UHZ. Immunostaining revealed markedly higher levels of BiP in HCs of the LHZ than in wt littermates (Figure 3E). Immunoblot analysis of proteins extracted from hypertrophic cartilage confirmed this up-regulation of BiP in 13del mice (unpublished data).

Another bZIP transcription factor, ATF4, regulates ER stress by inducing the apoptosis-associated gene *Chop* [30]. Quantitative RT-PCR showed marked up-regulation of *Chop* transcript (unpublished data), and immunostaining showed elevation of CHOP protein in 13del HCs (Figure 3F) compared to wt mice, but not of apoptosis as determined by terminal transferase dUTP nick end labeling (TUNEL) assays (Figure S4). Cytoplasmic localization of p53 is a molecular consequence of preventing apoptosis during ER stress [31], and we observed this in 13del HCs in the LHZ (Figure 3G). The ER-stressed HCs survived, which is consistent with an expansion of the HZ.

### Normal Rate of Chondrocyte Hypertrophy in 13del Proliferating Chondrocytes

**Figure 4:**
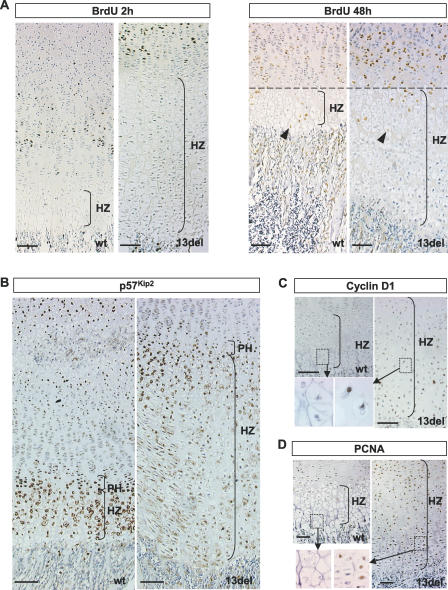
Abnormal Cell-Cycle Regulation in 13del HCs (A) BrdU pulse/chase assay. Left panel; 5-d-old mice were injected with a single dose of BrdU and sacrificed 2 h later. Right panel; 8-d-old mice received two pulses of BrdU with an intervening interval of 6 h and were sacrificed 48 h after the first injection. The rate of chondrocyte hypertrophy is similar in wt and 13del mice; the most distally located BrdU-labeled cells (arrowheads) occurred at the same level relative to the onset of the HZ. (B–D) Immunohistochemical detection of p57^Kip2^ (B), cyclin D1 (C), and PCNA (D). The boxes in (C and D) indicate the regions of higher magnification shown in the bottom left corner. Color contrast of the low-power magnification images in (A–D) were adjusted as described in Materials and Methods. Bar indicates 100 μm.

To determine whether the accumulation of HCs was due to increased rate of chondrocyte hypertrophy, we performed in vivo pulse-labeling experiments using 5-bromo-2′-deoxyuridine (BrdU). Two hours after BrdU injection, only proliferating chondrocytes were labeled in both 13del and wt mice (Figure 4A). There was no difference between wt and 13del in the proportion of labeled proliferating cells in the proliferating zone (PZ) (11.17% and 10.65%; *p* = 0.116; *n* = 3 for each genotype). After a 48-h chase, the position of the most distally located BrdU-labeled HCs relative to the PH was similar in 13del and wt mice, indicating that the transition to hypertrophy was unaffected (Figure 4A). The percentage of BrdU-labeled HCs relative to total number of BrdU-labeled chondrocytes in the PZ and HZ was also similar (*p* = 0.53) in 13del (31.9 ± 3.8%; *n* = 4) and wt mice (30.7 ± 4.4%; *n* = 4), indicating that the rate of chondrocyte progression to the hypertrophic state is normal in 13del. This suggests that the 13del-induced defect arises within the HZ.

### Altered Cell-Cycle Regulation in 13del HCs

p57^Kip2^ regulates cell-cycle exit and postmitotic hypertrophic differentiation in chondrocytes [32,33]. Expression of p57^Kip2^ in the UHZ was up-regulated to a similar degree in 13del and wt mice (Figure 4B), which indicates that 13del cells had exited the cell cycle and entered the hypertrophic program. However, p57^Kip2^ was significantly down-regulated in the mid to lower 13del HZ (Figure 4B), which suggests that the cells could have re-entered the cell cycle.

The 13del expressing HCs in the mid to lower HZ, but not wt cells, were also found to express cyclin D1, a marker for the G1 phase (Figure 4C, Figure S3B). Proliferating cell nuclear antigen (PCNA), a marker for proliferating cells, was also expressed specifically by cells of the 13del HZ, but not wt (Figure 4D). Together, these data suggest that in 13del mice, hypertrophic differentiation was initiated, but HCs in the mid to lower HZ re-entered the cell cycle. However, given that BrdU-labeled HCs were not detectable in the 13del HZ after 2 h (Figure 4A), 13del HCs did not appear to have progressed through the G1/S checkpoint. Thus, expansion of the HZ cannot be attributed to the proliferation of HCs by mitotic division.

### Re-expression of Gene Markers for Earlier Chondrocytic Lineages by 13del HCs

We observed that some chondrocytes within the 13del LHZ acquired a more flattened and elongated cell shape with condensed nuclei (Figure 4B–4D). Moreover, we found that there were increased numbers of smaller chondrocytes in the 13del LHZ close to the chondro-osseous junction (29.5% ± 6.42%) compared to wt LHZ (17.8% ± 7.49%) (*p* = 0.04 by *t*-test; see Materials and Methods), indicating a change in identity. Thus, we hypothesized that the differentiation status of HCs in the HZ had been altered, contributing to the expansion of the HZ. The different subpopulations of chondrocytes display characteristic expression profiles of many genes encoding transcription factors (e.g., *Sox9*), signaling/growth factors (e.g., *Ihh, Pthrp,* and *Igf2*), receptors (e.g., *patched [Ptc]* and *PTH/PTHrP receptor [Ppr]),* cell-cycle regulators (e.g., *p57^Kip2^* and *cyclin D1*), and ECM components *(Col2a1, Col10a1,* and *Agc1)*.

**Figure 5:**
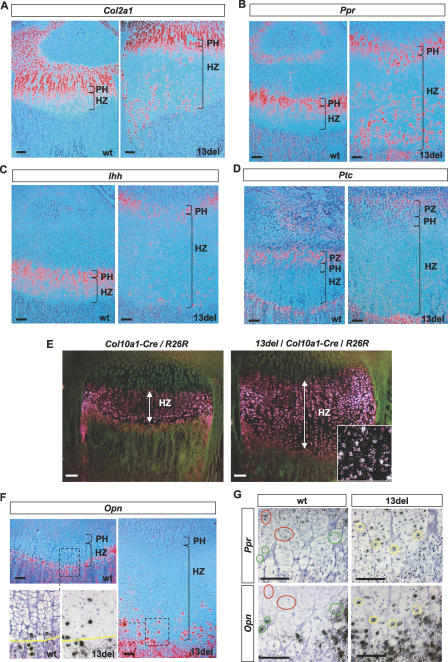
Altered Differentiation of HCs to “PreHC-Like” Cells Analyses of the proximal tibial growth plate of 10-day-old mice. (A–D and F) In situ hybridization using specific markers for resting, proliferating chondrocytes, and preHCs *(Col2a1)* (A), preHCs *(Ppr)* (B), preHCs and HCs *(Ihh)* (C), proliferating chondrocytes *(Ptc)* (D), and terminal HCs *(Opn)* (F). The chondro-osseous junction is depicted by a yellow line). (E) To show that altered differentiation took place after the initiation of hypertrophy, X-gal staining was performed on 10-d-old *Col10a1-Cre*/ROSA26 Cre reporter (R26R) mice with or without the *13del* transgene. Positive staining appears as pink under dark field. The inset in the 13del panel shows co-localized X-gal staining (pink) and *Ppr* in situ hybridization signal (white dots) in mid-lower HZ of the triple mutant. (G) In situ hybridization for *Opn* and *Ppr* in consecutive sections. In some HCs of 13del mice, *Opn* and *Ppr* are co-expressed (yellow-circled cells). In wt mice, the expression profiles of *Opn* and *Ppr* are mutually exclusive (see red- and green-circled cells). Bar indicates 100 μm.

As wt proliferating chondrocytes undergo hypertrophy, several genes are down-regulated in preparation for replacement of cartilage by bone. *Col2a1* is normally expressed throughout the growth plate with the highest levels in the resting zone (RZ), PZ, and PH, and the lowest in the HZ (Figure 5A). *Sox9* expression parallels that of its transcriptional target *Col2a1,* except in the HZ, where it is absent (Figure S5A). *Igf2* is expressed at high levels in proliferating chondrocytes and at lower levels in preHCs, and is down-regulated in HCs (Figure S5B). Consistent with the finding that entry into the hypertrophic phase was not affected in 13del mice, the expression of *Col2a1, Sox9,* and *Igf2* was down-regulated in the UHZ. However, 13del chondrocytes in the mid- and lower portions of the HZ re-expressed *Sox9, Col2a1,* and *Igf2* (Figures 5A, S5A, and S5B). The α1-chain of collagen I *(Col1a1),* a marker of osteoblasts, was not expressed in 13del HCs (Figure S5C). Because HCs in the LHZ have normally progressed to the terminal stages of differentiation, our data indicate 13del LHZ chondrocytes had reverted to a “preHC-like” status.

### Re-Activation of the IHH and PPR Signaling Pathways in 13del HCs

To further characterize the abnormality in terminal chondrocyte differentiation, we focused on two pathways, IHH/PTC and PTHrP/PPR, which cooperate to regulate chondrocyte proliferation and hypertrophy [34]. In 10-d-old wt mice, expression of *Ppr* (encoding PTH/PTHrP receptor) and *Ihh* was found predominately in both preHCs and upper HCs (Figure 5B and 5C), and *Ihh* mRNA was diminished in the LHZ and absent from cells bordering the chondro-osseous junction. Expression of *Ptc,* which encodes for IHH receptor and a target gene of IHH per se, flanked the *Ihh*-expressing domain proximally and distally, just adjacent to the preHCs and within the osteogenic cells at the chondro-osseous junction (Figure 5D). In 10-d-old mice, *Ihh, Ptc,* and *Ppr* were expressed in similar regions in both 13del and wt; but in 13del mice, these genes were re-expressed in some cells in the LHZ (Figure 5B–5D). The strong activation of *Ptc* transcription and re-expression of *Ppr* indicate that cells in the 13del LHZ are receiving and responding to IHH signal. Overall, 13del cells in the LHZ expressed markers that are more characteristic of preHCs.

### Altered Terminal Differentiation in 13del HCs

To confirm that 13del HCs had initiated hypertrophic differentiation normally, we created *Col10a1-Cre*/ROSA26 CRE reporter mice that expressed CRE recombinase and β-galactosidase specifically in HCs and crossed them with 13del mice. In the compound mutant mice with or without *13del* transgene, all the HCs expressed β-galactosidase as shown by X-gal staining (Figure 5E). Some 13del HCs in the mid-lower HZ exhibited both *Ppr*-re-expression and positive X-gal staining (Figure 5E, inset in 13del panel). Because the *Cre* gene had been inserted into the *Col10a1* gene by homologous recombination, its expression is under the control of the endogenous *Col10a1* promoter. Therefore, the induction of β-galactosidase expression indicated that differentiation of 13del HCs was disrupted after they have initiated hypertrophy.

As HCs attain the terminal stages of differentiation at the chondro-osseous junction, they express *osteopontin (Opn)* and *matrix metalloproteinase 13 (Mmp13)*. In wt mice, these genes were expressed exclusively in HCs at the chondro-osseous junction, whereas in 13del mice, expression of *Opn* and *Mmp13* (Figure 5F and Figure S5D) were delayed and scattered throughout the LHZ. In situ hybridization of consecutive sections through 13del growth plates revealed that some ectopic *Opn*-expressing HCs also expressed *Ppr,* manifesting the characteristics of both preHC and terminally differentiated chondrocytes (Figure 5G). This co-expression of *Opn* and *Ppr* was not observed in wt growth plates (Figure 5G).

### Altered Differentiation and Function of 13del HCs Is Cell Autonomous

**Figure 6:**
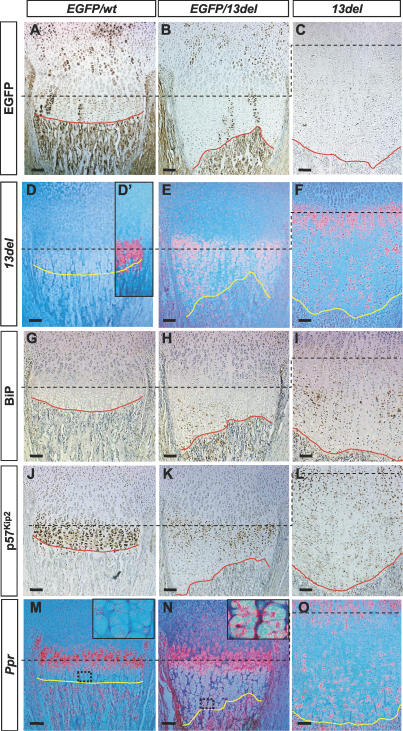
The Signal for the Altered Differentiation of 13del HCs Is Cell Autonomous Analyses of EGFP/wt and EGFP/13del chimeras. (A, B, and C) In EGFP/13del chimeras, normal (wt) cells express EGFP. (D, E, and F) 13del cells are identified by in situ hybridization with a 64-bp probe specific for the *13del* transcripts. (D′) shows expression of *Col10a1* in the normal EGFP/wt chimera (consecutive section). (G–I) Immunohistochemical detection for BiP. (J–L) Immunohistochemical detection for p57^Kip2^. (M–O) In situ hybridization for *Ppr*. The insets in (M) and (N) show regions in the LHZ containing wt and 13del cells, respectively, at higher magnification. (N) shows marked re-expression of *Ppr* in 13del cells. Chondro-osseous junctions are traced using a red or yellow line. Color contrast of (A–C) and (G–L) was adjusted as described in Materials and Methods. Bar indicates 100 μm.

To determine whether the altered differentiation program was intrinsic to 13del chondrocytes, we created mouse chimeras by aggregating morulae from 13del mice and from mice ubiquitously expressing the enhanced green fluorescent protein (EGFP). Wt cells were detected by immunostaining for EGFP (Figure 6A–6C); 13del cells were detected by in situ hybridization using the 13del-specific riboprobe (Figure 6D–6F). Mice with different degrees of EGFP/13del chimerism, as well as EGFP/wt chimeras, were studied and expression of BiP (Figure 6G–6I), p57^Kip2^ (Figure 6J–6L) and *Ppr* (Figure 6M–6O) were analyzed.

As anticipated, the growth plates of EGFP/wt chimeras were indistinguishable from wt (Figure 6A). In EGFP/13del chimeras, the most noticeable changes were variations in the height of the HZ and an undulating chondro-osseous junction in the growth plate (Figure 6B). Where the contribution of 13del cells was higher, the degree of expansion of HCs was likewise higher and the undulation of the chondro-osseous junction generally correlated well with the relative contribution of 13del cells. This correlation suggests that the defect in 13del HCs is a cell-autonomous one, since their abnormal differentiation could not be overcome by the presence of wt HCs.

Key marker for ERSS (BiP) (Figure 6H), for cell-cycle progression (p57^Kip2^) (Figure 6K), and for chondrocyte differentiation *(Ppr)* (Figure 6N) were expressed abnormally in regions containing 13del cells. 13del HCs in the LHZ of chimeras re-expressed *Ppr* (Figure 6N). Thus, the presence of wt cells did not “rescue” the 13del cells, and the signal for altering differentiation program is most likely cell autonomous.

### 13del Expression Correlates with the Levels of ER Stress and Alteration in Differentiation

**Figure 7:**
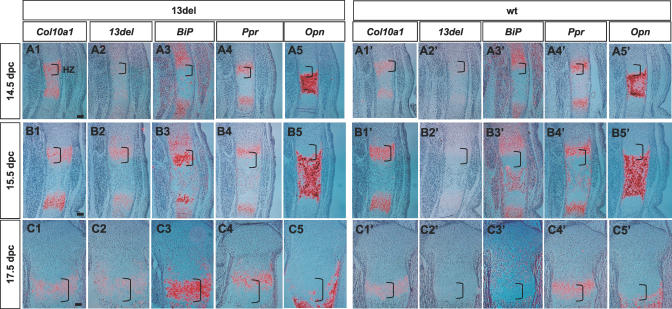
The Dynamics of Transgene and *BiP* Expression, HZ Expansion, and Altered Differentiation during Prenatal Growth Gene expression analyses on the proximal tibial growth plates from 14.5 (A1–A5 and A1′–A5′), 15.5 (B1–B5 and B1′–B5′), and 17.5 dpc (C1–C5 and C1′–C5′) mice. In situ hybridization was performed on paraffin sections using probes detecting *Col10a1* (first column in both 13del and wt sections), *13del* (second column in each section), *BiP* (third column in each section), *Ppr* (fourth column in each section), and *Opn* (fifth column in each section). Brackets indicate HZ. Bar indicates 100 μm.

**Figure 8:**
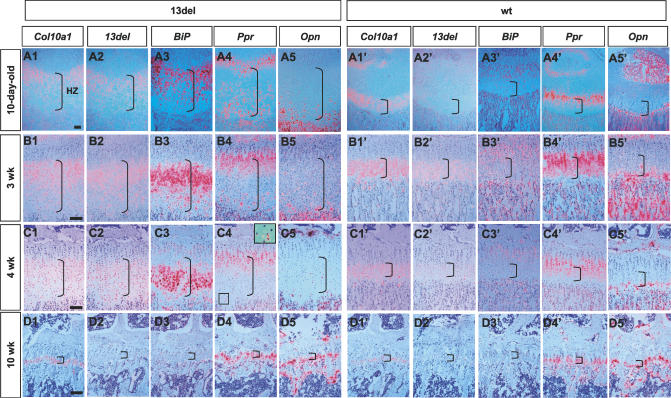
The Dynamics of Transgene and BiP Expression, HZ Expansion, and Altered Differentiation during Postnatal Growth Gene expression analyses on the proximal tibial growth plates from 10-d-old (A1–A5 and A1′–A5′), 3-wk-old (B1–B5 and B1′–B5′), 4-wk-old (C1–C5 and C1′–C5′), and 10-wk-old (D1–D5 and D1′–D5′) mice. In situ hybridization was performed on paraffin sections using probes detecting *Col10a1* (first column in both 13del and wt sections), *13del* (second column in each section), *BiP* (third column in each section), *Ppr* (fourth column in each section), and *Opn* (fifth column in each section). The insets in (C4) shows a region (marked by broken lines) in the LHZ at higher magnification, containing 13del cells that re-expressed *Ppr*. Brackets indicate HZ. Bar indicates 100 μm.

To understand the relationship between *13del* transgene expression, ERSS, and the alteration in differentiation in fetal and postnatal growth, expression of the *13del* transgene, the endogenous *Col10a1* gene, *BiP,* and *Ppr* was followed over fetal and postnatal stages. Interestingly, at 14.5 dpc, although expression of endogenous *Col10a1* (Figure 7A1) can be seen in the HZ of 13del mice, little or no *13del* transgene expression was detected (Figure 7A2), and *BiP* expression was not induced in the HZ (Figure 7A3). Similar to wt mice, expression of *Ppr* was restricted to the PH and upper HZ in 13del mice and no expansion of HZ was detected (Figure 7A4 and 7A4′). In addition, *Opn* expression was restricted to the last layer of HCs and the primary ossification center in both genotypes (Figure 7A5 and 7A5′). *13del* transgene expression in HCs could be detected at 15.5 dpc (Figure 7B2), with a concomitant induction of strong expression of *BiP* in the HZ (Figure 7B3; in contrast to Figure 7B3′). At this stage, the HZ in 13del was not expanded, and *Ppr* and *Opn* expression patterns were normal (Figure 7B4 and 7B4′, and 7B5 and 7B5′). At 17.5 dpc, the expression pattern of these key genes remained similar; however, a slight expansion of the HZ and punctate *Opn* expression in the LHZ could be detected in 13del mice (Figure 7C1-7C5). Re-expression of *Ppr* in the LHZ was detectable at 18.5 dpc (unpublished data), and most obvious at 10 d old (Figure 8A4) that correlated with the greatest HZ expansion (Figure 8A1-8A5) and induction of *BiP* expression (Figure 8A3). The *13del* transgene, *Col10a1,* and *BiP* expression were down-regulated in the LHZ of 13del mice at 10 day old, when *Ppr* was re-expressed (Figure 8A1-8A5) and *Opn* showed punctate expression (Figure 8A1-8A5). A similar pattern for these markers was also observed in 3-wk-old 13del mice, although the degree of HZ expansion was reduced in terms of the number of cell layers in the HZ (Figure 8B1-8B5).

In 4-wk-old 13del mice, expression of the *13del* transgene was relatively weaker than endogenous *Col10a1* and was distributed through the middle to lower HZ (Figure 8C2 and 8C2′). Expansion of HZ was further diminished; strong expression of *BiP* persisted only in the middle to lower HZ (Figure 8C3), and there were much fewer cells expressing *Ppr* in the LHZ (Figure 8C4). By 6 wk and 10 wk, *13del* transgene expression continued to diminish, being undetectable by 10 wk (Figure 8D2 and unpublished data), although robust levels of the endogenous *Col10a1* gene were found (Figure 8D1). Induction of *BiP* was barely detectable at 6 wk and was absent at 10 wk (Figure 8D3 and unpublished data). These were accompanied with return to normal height of HZ and normal *Ppr* and *Opn* expression pattern similar to wt (Figure 8D4 and 8D5 and unpublished data).

**Figure 9:**
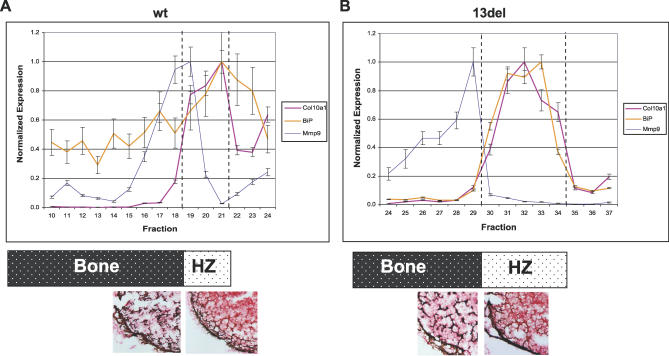
Correlation of Altered Differentiation of HCs and ERSS Serial transverse sections of 10-d-old tibiae were collected for quantitative RT-PCR analyses. A section between each fraction of 20 sections was stained with safranin-O and the von Kossa stain to identify the bony or cartilaginous nature of each fraction. The section around the chondro-osseous junction is shown from wt (A) and 13del (B) mice. Mineralized tissues stained dark brown and cartilaginous tissues stained orange. Relative transcript abundance was quantified for *Col10a1* and *13del*, *BiP,* and *Mmp9* in wt (A) and 13del (B). The *Col10a1* primers also recognize *13del* transcripts. Fractions are numbered from the bone shaft towards the growth plate cartilage for reference only, and there is no correlation between wt and 13del fractions. The position of the fractions relative to the bone and HZ of the growth plate is indicated. The HZ is delineated by a pair of dotted line in each graph; PZ and RZ are on the right-hand side of the HZ. Error bars represent ±1 standard deviation (SD).

The close correlation between *Col10a1* and *13del* expression with BiP was further shown by quantitative RT-PCR using serial transverse sections of the tibial growth plates. In the wt, levels of *Col10a1* were high throughout the HZ, decreasing sharply across the chondro-osseous junction (Figure 9A, fraction 19–18). This reduction in expression is normally accompanied by an up-regulation of *Mmp9,* a marker for terminally differentiated HCs (Figure 9A, fraction 19–18). *BiP* expression was low throughout, and the levels showed only mild fluctuation. In the 13del HZ, *Col10a1* and *13del* levels were high in the UHZ. The up-regulation of *Col10a1* and *13del* expression was closely correlated with strong induction of *BiP* and *Chop* expression (Figure 9B, Fraction 34–30; unpublished data for *Chop*). In the 13del LHZ, *Col10a1* and *13del* expression was down-regulated some distance away from the chondro-osseous junction, which is marked by *Mmp9* up-regulation (Figure 9B, fraction 31–29). This down-regulation was accompanied by reduced *BiP* and *Chop* expression in the LHZ, indicating ER stress was alleviated (Figure 9B, fraction 31–30). These data suggest that ER stress and its alleviation are closely associated with the altered terminal differentiation in 13del HCs.

Together, these data demonstrated that in 13del mice, transgene expression activated ERSS in the first instance, followed by *Ppr* re-expression in the LHZ, leading to an expanded HZ. From weaning age (3 wk) onwards, transgene expression level steadily diminished together with attenuation of ERSS.

### ER Stress in Other Mouse Models of Chondrodysplasia

**Figure 10:**
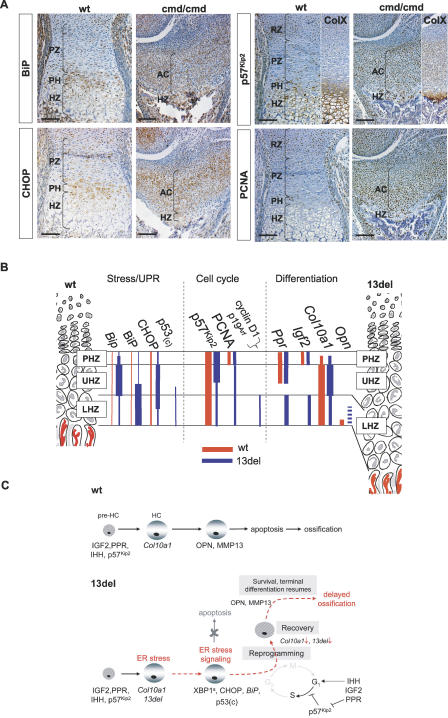
ERSS in *cmd/cmd* Mutant and a Model for Surviving ER Stress (A) ERSS and disrupted chondrocyte differentiation in the growth plate of the *cmd/cmd* mutant. Immunohistochemical detection of BiP, P57^Kip2^, PCNA, and collagen X (ColX) in the tibial growth plates of 18.5 dpc fetus. Color contrast of these images was adjusted as described in Materials and Methods. Bar indicates 100 μm. (B) Comparative summary of expression pattern for molecular markers in the growth plate in wt and 13del mice. (C) Model for the reprogramming of HCs in 13del transgenic mice expressing unfolded collagen X. AC, zone containing abnormal chondrocytes; p53(c), cytoplasmic localized p53.

To assess whether the ERSS can be induced in chondrocytes at a different stage of differentiation and is, therefore, potentially implicated in other forms of chondrodysplasia, we tested whether ER stress was triggered in the chondrocytes of growth plates of two other mouse models of chondrodysplasia. *cmd/cmd* mutants express a severely truncated aggrecan core protein because of a premature stop codon in exon 5 [35]. *Col2a1^G904C^* transgenic mice express procollagen II with a glycine to cysteine mutation (G904C) [36]. Both mutants show a fragmented ER in chondrocytes (*Col2a1^G904C^,* Figure S6; *cmd/cmd* mice [37]). The normal chondrocyte columns were disorganized, and immunostaining for BiP was enhanced in both mutants, indicating induction of ERSS (Figure 10A, Figure S6). There was no distinct zone of proliferating chondrocytes in the *cmd/cmd* mutant, with only a very small portion of cells progressing to hypertrophy, indicated by the region of the growth plate stained for collagen X (Figure 10A). *CHOP* expression was elevated, but there was no increase in apoptotic cells (unpublished data). There was also increased ectopic expression of cell-cycle markers such as p57^Kip2^ and PCNA (Figure 10A), indicating these cells are trapped in an abnormal state of cell-cycle progression.

## Discussion

Many studies have shown that factors influencing cell fate and/or differentiation are activated in ERSS, but how such changes impact differentiation programs in vivo is poorly understood. We have shown that HCs in the murine growth plate expressing unfolded collagen X experience ER stress but do not undergo apoptosis. The normal terminal differentiation process is interrupted; HCs adapt and survive via cell-autonomous reversion to a preHC-like state that results in delayed endochondral ossification and chondrodysplasia.

### Expression of Unfolded Collagen X Triggers ER Stress

In vitro translation assays suggest that 13del mutant collagen X proteins are unable to assemble into trimers, and thus are poorly secreted. The accumulation of the 13del proteins in the ER and the low to undetectable level in the ECM of the HZs of 13del mice confirmed the secretion defect. Poor secretion is also seen with collagen X containing other MCDS mutations that prohibit subunit folding and assembly [23,38].

The 13del mice reveal that misfolding of mutant collagen X occurs in vivo. High-level expression of *Col10a1* is characteristic of chondrocyte hypertrophy. 13del HCs transcribed both endogenous *Col10a1* and *13del* transgene. Intracellular accumulation of 13del proteins suggested that the endogenous degradation machinery could not clear all the mutant protein. The distended, fragmented ER indicates that ER stress was induced in HCs expressing 13del proteins, leading to activation of ERSS. Up-regulation of *BiP, CHOP,* expression of *Xbp1^s^, Edem,* and cytoplasmic localization of p53 in 13del HCs are all indicators that ERSS was triggered. The co-expression of both XBP1^s^ protein and 13del in the same cells suggests that expression of the mutant collagen directly induced ERSS.

Interestingly, in contrast to the expanded HZ in 13del mice, expression of chicken collagen X chains containing a deletion within the helical domain in transgenic mice results in chondrodysplasia and a compressed HZ [39]. The underlying molecular mechanism is not clear, and there are no mutations reported in the helical domain of collagen X in humans for comparison. Trimerization of the mutant chicken collagen X was not affected and homo- and hetero-trimers formed efficiently in vitro [40]. Furthermore, mutant collagen X is detected in the extracellular matrix in growth plate cartilage of the transgenic mice, with little evidence for intracellular accumulation [40]. Thus, the molecular consequence to 13del mice is likely to be different, and the compression may be related to the presence of mutant chicken collagen X mixed with wt mouse collagen X or hybrid mouse–mutant chicken collagen X in the growth plate matrix, impairing the supramolecular structure of extracellular collagen X and hence tissue integrity.

### Visualizing Induction and Progression of ERSS in the 13del Growth Plate In Situ

A single histological section of the growth plate provides a continuous snapshot of the different phases of chondrocyte differentiation as they progress from a resting state to the proliferative state, become prehypertrophic, and then enter the final stages of hypertrophy and terminal differentiation at the chondro-osseous junction. Thus, our analyses of molecular changes in 13del chondrocytes using longitudinal sections taken through the growth plate provide an in vivo picture of ER stress induction. The response of the HCs to ER stress, indicated by induction of *BiP* expression, can then be monitored as the HCs approach the chondro-osseous junction. In cultured cells, induction of ERSS is characterized by a rapid increase of *BiP* mRNAs [41,42].

In 13del, strong induction of *BiP* mRNA in the UHZ was observed in pre-weaning age, suggesting that HCs are experiencing ER stress soon after hypertrophy. In contrast, by 4 wk postpartum, in UHZ chondrocytes, *13del* transgene expression was diminishing, whereas *BiP* mRNA was more abundant in the LHZ where cells were expressing a higher level of the transgene and ER stress is maintained in these cells. Therefore, the stress level in 13del HCs varied during growth according to transgene expression level, and the stress response is dosage dependent.

It is interesting to note that the *13del* transgene did not fully recapitulate the expression of the endogenous *Col10a1* gene in fetal and postnatal growth. It is turned on later (between 14.5 and 15.5 dpc) and turned off earlier (between 6–10 wk postpartum) when compared to the endogenous *Col10a1,* probably because the transgene did not contain the full complement of *Col10a1* regulatory elements; for example, the lack of a conserved enhancer element upstream of the transgene ([43] and unpublished data). When normal *Col10a1* alone was expressed (14.5 dpc and at 10 wk postpartum), induction of *BiP* expression was not detected. This asynchrony in timing of expression between *13del* and the endogenous *Col10a1* and the qRT-PCR data serves to highlight the close association between *13del* expression and *BiP* up-regulation, further supporting the notion that 13del proteins induced ERSS directly.

### Surviving ER Stress Is Coupled with Altered Terminal Differentiation

ERSS is primarily a response to relieve ER stress for cell survival. However, ER stress can lead to cell death as shown in various systems. In the Akita diabetic mouse, ER stress led to apoptosis via *CHOP* induction. In the absence of CHOP, apoptosis was reduced and the onset of diabetes was delayed [15,44]. CHOP is also implicated for anti-apoptotic activity. In a mouse model of human Pelizaeus-Merzbacher disease experiencing ER stress, CHOP protects oligodendrocytes from apoptosis [7]. These contradictory results may be explained by the dependence of concurrent signaling events during ER stress. Indeed, signals both upstream and downstream of CHOP (i.e., the PERK signaling cascade) have been shown to have opposite effects on cell survival [45–47]. In 13del, despite raised CHOP expression in the UHZ, apoptosis was not triggered and the HCs survived. In this situation, ERSS may play a cytoprotective role. It is known that growth plate chondrocytes are destined to survive hypoxic stress during development [48]. This implies that they are preconditioned to survive hypoxic conditions before becoming terminally differentiated, probably through the cytoprotective actions of HIF-1α PERK, and eIF2α [6,49,50]. Chondrocytes may have additional adaptive mechanisms that protect against stress-induced apoptosis or have a high stress tolerance or threshold before cell death can be triggered. One may speculate that cells that are not preconditioned to survive stress (e.g., neurons) may be much more susceptible to ER stress-induced death. Paradoxically, the cytoprotective effect of ERSS may interfere with the normal apoptotic fate of HCs, contributing to the abnormal terminal differentiation of 13del HCs.

Survival of 13del HCs was associated with signs of stress alleviation. The diminished expression of *Col10a1/13del* in the LHZ indicates that production of the mutant collagen X chains in those chondrocytes was reduced, and ER stress was correspondingly alleviated, as revealed by the down-regulation of *BiP* and *Chop* transcript in the LHZ. The outcome of alleviation of ER stress is the reduction in synthesis of 13del proteins. In 13del, a reduction in *Col10a1* and *13del* mRNAs occurred that must contribute towards alleviation of ER stress. Such reduction could occur by nonsense-mediated decay, as suggested for MCDS caused by the nonsense mutations [51,52] or by IRE1-mediated mRNA degradation recently identified to occur during the UPR [53] or by regulating transcription.

It is notable that the reduction in *Col10a1/13del* expression also correlated with the change in cell-cycle control and differentiation state of the chondrocytes in the LHZ (summarized in Figure 10B). These changes point to more complex modes of alleviating ER stress. In the 13del UHZ, the strong transcription of both wt and 13del *Col10a1,* up-regulated expression of p57^Kip2^, and extent of BrdU labeling in pulse-chase experiments indicated that 13del preHCs had exited the cell cycle and progressed into hypertrophy normally. Normal entry into hypertrophy is further confirmed by the expression of *Col10a1-Cre* and β-galactosidase activity in all HCs in the 13del expanded HZ. However, down-regulation of both *Col10a1* and *13del* transcripts in advance of expression of *Mmp9,* and delayed expression of *Mmp13* and *Opn* (relative to the onset of hypertrophy) in the 13del LHZ, indicated that terminal differentiation was disrupted.

In the 13del LHZ chondrocytes, re-expression of genes *(Ihh, Ppr, Ptc, Igf2, Sox9,* and *Col2a1)* characteristic of proliferating chondrocytes/preHCs indicated a switch to preHC-like status. In addition, the down-regulation of p57^Kip2^ and the up-regulation of PCNA and cyclin D1 are consistent with cell-cycle re-entry. This is a novel finding since HCs are terminally differentiated and are not expected to return to the cell cycle. PTHrP suppresses *p57^Kip2^* expression [33], IGF2 antagonizes *p57^Kip2^* [54], and cyclin D1 is a target of IHH [55] and IGF2 [56] signaling. *Igf2, Ppr,* and *Ihh* show highest expression level in wt proliferating chondrocytes or preHCs, and are down-regulated in HZ. Their re-expression in the 13del LHZ suggests a link to the observed cell-cycle re-entry. Activated PTHrP and IGF2 signaling may down-regulate p57^Kip2^ expression, whereas activated IHH and IGF2 signaling could up-regulate cyclin D1 expression, stimulating cell-cycle re-entry. Cell-cycle re-entry for 13del LHZ chondrocytes is most likely a secondary effect due to the re-activation of signaling pathways that regulate chondrocyte proliferation and hypertrophy.

Cells in the 13del LHZ were asynchronized in terms of their differentiation state: some of them expressed preHC markers (e.g., *Ppr*), some expressed markers for terminally differentiated HCs (e.g., *Opn*), and some expressed both *Ppr* and *Opn*. The latter can be interpreted as cells captured in a “reprogramming” process. Because, as indicated in wt mice, expression of *Opn* marks HCs that have progressed to the terminal status, and for *Ppr* to be expressed in the same 13del HCs suggests a reversion process has occurred. However, the reversal of differentiation is not absolute, as endochondral ossification does occur, albeit delayed. Therefore, the co-expression of *Ppr* and *Opn* could also indicate stress-alleviated HCs resuming terminal differentiation. These cells may bypass the intermediate hypertrophic state to avoid expressing high level of mutant collagen X.

### Relationship between ER Stress and Altered Terminal Differentiation

The inability of wt HCs to rescue the delayed terminal differentiation, and the altered gene expression of 13del cells in chimeras, suggest that these changes are dominant and cell autonomous. The normal terminal differentiation of HCs in collagen X null mice [57] and identical expansion of HZ in compound *13del; Col10a1* null mutants (unpublished data) are consistent with such a dominant effect. Given that there is normal entry into hypertrophy and a clear demarcation region of fully differentiated HCs in the UHZ, as shown by the β-galactosidase activity in all HCs in *Col10a1-Cre*–Rosa 26 reporter mice, the reversion of cells in the LHZ to a preHC-like status must be due to altered terminal differentiation of the stressed HCs.

The concomitant onset of ERSS in the 13del UHZ with disruption of terminal differentiation provides visual evidence for a possible link between these two processes, and is consistent with the latter occurring as a consequence of ERSS. The correlation between levels of 13del expression with degree of HZ expansion in independent lines of mice suggests a direct link between level of ER stress and the altered differentiation. In addition, the gradual loss of preHC-like cells after weaning age correlates with the down-regulation of *13del* and delayed *BiP* induction, suggesting a causal relationship between ER stress and altered HC differentiation. Co-expression in the same cells of 13del with XBP1^s^ and with cyclin D1 proteins provides molecular evidence for a direct link.

Reverting to a preHC-like state may be an adaptive or fortuitous response by which the 13del HCs alleviate and survive ER stress. This is interesting, since by reverting to a preHC-like state, both wt and 13del *Col10a1* transcription would be down-regulated, thereby reducing the source of the ER stress (13del proteins) to a more acceptable level. Consistent with this hypothesis, expression of *13del, Col10a1,* and *BiP* was reduced in the LHZ at 10 d and 3 wk postpartum.

Chondrocytes in culture are able to re-differentiate after dedifferentiation to a fibroblastic/osteoblastic state [58,59]. This plasticity involves changes in transcriptional profile and may be retained in HCs. The altered terminal differentiation of 13del HCs is the first in vivo demonstration of the phenotypic plasticity and capacity to revert to a preHC-like state. Unlike in cell culture, none of the 13del HCs expressed *Col1a1,* the osteoblast marker, suggesting that reprogrammed differentiation to a preHC-like state occurred rather than transdifferentiation to osteoblasts. This inherent plasticity may be exploited by HCs to adapt and survive ER stress in vivo.

Our findings provide novel mechanistic insight into how chondrocytes overcome ER stress in vivo. Until now, it is generally accepted from in vitro studies that cells respond to ER stress either by undergoing apoptosis or by a general down-regulation of protein synthesis and up-regulation of protein-folding/degradation capacity and thereby relieve the load of unfolded proteins in the ER. However, there is no information on the impact of ER stress on the differentiation program of chondrocytes either in vitro or in vivo.

We propose a model (Figure 10C) in which, in addition to the alleviation mechanisms mediated by the UPR, 13del HCs cope with ER stress through a “reprogram, recover, and survive” adaptive mechanism. Initially, 13del protein expression induces ERSS, but not apoptosis. The differentiation program of the HCs is altered in which they “revert” or “reprogram” to a preHC-like state in which collagen X expression is reduced. The “reprogramming” involves re-activation of signaling pathways that may stimulate re-entry into the cell cycle. However, p57^Kip2^ restricts cell-cycle progression. By reprogramming, the 13del HCs finally down-regulate endogenous *Col10a1* and *13del* mRNA levels, thereby alleviating the load of mutant protein and ER stress, providing the means to survive and complete differentiation, albeit with delayed endochondral ossification.

The exact mechanisms that mediate the reversion of the differentiation pathway in 13del HCs are not known. It is conceivable that the ER stress response takes control over gene expression and protein metabolism to such a significant extent that it interferes with normal differentiation processes, which may involve complex alteration in signal transduction and transcription factors recruitment and interaction, as well as epigenetic changes upon ER stress [60]. Identifying and understanding the underlying mechanisms are important and issues for further study.

### Implications for the Pathogenesis of Chondrodysplasias

Our data have broad implications for the mechanism of disrupted chondrocyte differentiation in MCDS and other chondrodysplasias caused by mutations that impair protein assembly and secretion. Although ER stress-induced apoptosis may provide a major route for pathogenesis, our study raises the possibility that chondrocytes can survive ER stress in a process that changes the normal differentiation program. Changes in the pattern of markers that characterize the differentiation status of chondrocytes have been noted in chondrodysplastic models. Mutations in collagen II that interfere with assembly and secretion, leading to chondrodysplasia, are associated with distension of the ER [61,62]. In *Col2a1^G904C^* mice, the fragmented ER, as well as the increased levels of BiP and CHOP in growth plate chondrocytes, indicate that ERSS is induced; abnormal terminal differentiation of HCs is indicated by the up-regulation of *Col2a1* and *Agc1* expression [36]. Transgenic mice expressing collagen II with a 36 amino acid deletion showed disorganized chondrocytes and fragmented ER, and there was loss of expression of the cell-cycle regulator *Cdkn1a* and the key differentiation markers *Ihh, Col10a1,* and *Fgfr3* [62]. In *cmd/cmd* mice, the distributions and co-expression patterns of several chondrocyte differentiation markers [63] and regulators of the cell cycle (p57^Kip2^ and PCNA) were altered. The abnormal gene expression in these models and 13del suggest that alteration of the normal progression of differentiation, cell-cycle control, and fate change may be a common consequence of ER stress in chondrocytes. However, the phenotypic consequence in terms of differentiation pathway and growth plate architecture may differ depending on the nature of the mutation and the expressing cell type.

The 13del mouse has also provided insight into the adaptive mechanisms that facilitate survival of chondrocytes in vivo in the face of ER stress. But survival is not without cost; the changes in HC differentiation do result in delayed endochondral ossification. We postulate that the change in differentiation program contributes to the disorganization of chondrocytes in the growth plate that occurs in many chondrodysplasias. Whether this strategy is peculiar to chondrocytes or can be adopted by other cell types affected in protein-folding disorders is an important issue to address in the future.

## Materials and Methods

### Generation of constructs and transgenic mice.

Thirteen nucleotides (residues 6058–6070) of the murine *Col10a1* gene were excised by overlapping PCR [22]. The resultant PCR product was cloned to generate the *Col10a1-13del* transgene. The transgene is a 10.5-kilobase (kb) fragment of the murine *Col10a1* gene containing 2 kb of the 5′ and 1.3 kb of 3′ flanking sequence after exon 3 (Figure 1B).

13del transgenic mouse founders were generated by pronuclear injection into one-cell CBA/C57BL6 F1-hybrid zygotes. Analyses were performed on 10-d-old littermates unless otherwise stated. Mice were genotyped by PCR using primers (5′-CCCAGGCATATACTATTTCTC-3′ and 5′-TAGCCTTTGCTGTACTCATC-3′) flanking the 13-bp deletion.

### Cell-free transcription and translation.

Wt and 13del full-length cDNA constructs in pBluescript II SK(−) (Stratagene, La Jolla, California, United States) were transcribed and translated using the TNT-T3 polymerase-coupled transcription and translation system (Promega, Madison, Wisconsin, United States), supplemented with canine microsomal membrane vesicles (Promega) as described previously [22]. For heterotrimer assembly, equal amounts of each of the normal and mutant plasmids were co-translated. Collagen X chains were analyzed on 7.5% (w/v) SDS-polyacrylamide gels.

### Histological and immunohistological analyses.

Limbs were fixed in 4% paraformaldehyde, and if necessary, were demineralized in 0.5M EDTA (pH 8.0) containing 0.2% formaldehyde prior to embedding in paraffin. Immunohistochemistry was performed using antibodies for p57^Kip2^, p53, cyclin D1, CHOP, PCNA (Santa Cruz Biotechnology, Santa Cruz, California, United States), XBP1^s^ (BioLegend, San Diego, California, United States), BiP (Stressgen, San Diego, California, United States), and green fluorescent protein (Abcam, Cambridge, United Kingdom). Signals were detected using the avidin-biotin-complex system (ABC; Vector Laboratories, Burlingame, California, United States) for p57^Kip2^, p53, and PCNA antibodies, or using the secondary antibody-HRP-conjugated polymer system (EnVision+; Dako, Glostrup, Denmark) for 13del, CHOP, BiP, cyclin D1; and green fluorescent protein antibodies. For XBP1^s^ and cyclin D1 antibody, signals were further amplified with biotinylated tyramide (PerkinElmer, Wellesley, Massachusetts, United States).

Immunostaining for collagen X was performed as described [57]. The 13del antibody was raised in rabbits against a synthetic peptide, AYTPLSMSTPLSQDS, which corresponds to the C-terminus of the 13del protein. Goat anti-rabbit Alexa 488 (Molecular Probes, Eugene, Oregon, United States) was used for immunofluorescence on cryosections. Concanavalin A conjugated with Alexa 594 (Molecular Probes) was used to visualize the ER.

### Histomorphometry and electron microscopy.

The vertical height of RZ, PZ, and HZ in the tibial growth plate was measured on tissue sections using a test line grid as described previously [64]. Sizes of cells within the tibial LHZ were measured in wt and 13del mice as described [65]. Measurements were made in cells in which the plane of section was through the nucleus to avoid bias. Cells were defined as being “smaller” if the maximal height was equal to or less than that of the maximal height of cells in the PZ of the same section. At least five sections from each animal for three wt and three 13del mice were analyzed. For electron microscopy, growth plate cartilage was embedded in Epon 812 and ultra-thin (60 nm) sections were prepared for electron microscopy analysis as previously described [66].

### BrdU incorporation.

Mice were injected intraperitoneally with 200 μg of BrdU per gram of body weight in either single dose (and sacrificed 2 h later) or two doses 6 h apart (and sacrificed 48 h after the first injection). Following fixation, BrdU in paraffin sections of limbs was detected using a BrdU Staining Kit (Zymed Laboratories, South San Francisco, California, United States). To determine the rate of hypertrophy, digital photographs were taken, and the number of BrdU-labeled chondrocytes in the PZ and HZ of the growth plate was counted. Cells in three consecutive sections from four 13del and four wt mice were scored; differences were assessed using the Student *t*-test.

### RNase protection assay and RT-PCR.

Total RNA was extracted from 10-d-old wt and 13del growth plate cartilage using Trizol reagent (Invitrogen, Carlsbad, California, United States), and RNase protection assays were performed using a 354-base [α-^33^P]UTP-labeled cRNA generated from the plasmid pWF36 (Figure 1B). Protected fragments were analyzed using 6% (w/v) polyacrylamide gel. RT-PCR was performed to amplify *Xbp1* mRNA residues 430–898 with specific primers. cDNA was synthesized from mRNA using SuperScript II (Invitrogen) primed with an antisense oligonucleotide (5′-GAGGTGCTTCCTCAATTTTCA-3′) against *Xbp1,* and used as a template in PCR for the amplification of *Xbp1* and a spliced variant, *Xbp1^s^,* using a sense primer 5′-GCTGGATCCTGACGAGGTT-3′ and the aforementioned antisense primer.

For quantitative RT-PCR, long bones from wt and 13del mice were collected and snap frozen in liquid nitrogen. The ends of the long bones were mounted in Tissue Freezing Medium (Jung, Nussloch, Germany) and growth plates fractionated by collecting 5-μm sections, 20 sections per fraction, on a Zeiss cryostat (Carl Zeiss, Oberkochen, Germany). In between each fraction, a single section was mounted and subjected to von Kossa and Safranin-O staining to determine cell morphology of the tissue comprising the adjacent fractions. Total RNA was prepared from growth plate fractions using the Trizol reagent. cDNA was prepared from fractions by reverse transcription of 1 μg of total RNA using Superscript II reverse transcriptase and random hexamers. Quantitative PCR was carried out on an ABI 7700 real-time thermal cycler using a SYBR green qPCR kit from ABGene. Specific PCR primers designed with a melting temperature (Tm) of approximately 60 °C and to span at least one intron were used to determine gene expression profiles. The primers used are as follows: *Col10a1,* 5′-TTCATCCCATACGCCATAAAG and 5′-AGCTGGGCCAATATCTCCTT; *BiP,* 5′-TGAAACTGTGGGAGGAGTCA and 5′-TTCAGCTGTCACTCGGAGAA; *Mmp9,* 5′-TTCGCGTGGATAAGGAGTTC and 5′-CCTCCACTCCTTCCCAGTCT; and *Actinb,* 5′-GACGGCCAGGTCATCACTAT and 5′-GTACTTGCGCTCAGGAGGAG. Dissociation curves were collected for all PCR reactions to ensure specificity. *Actinb* was used as an internal control for normalization. The highest expression level for a particular transcript was designated arbitrarily as 1 for comparison.

### In situ hybridization.

In situ hybridization was performed as previously described [63], using [^35^S]UTP-labeled ribopobes for *Ihh* (from A. McMahon), *PTHrP receptor (Ppr), Ptc, Opn,* and *Mmp13* (from H. Kronenberg), *Igf2* (from A. Ferguson-Smith), and *Edem* (from K. Nagata). To detect wt and 13del *Col10a1* mRNA, a 77-bp (nucleotides 1,824–1,899) wt-specific short probe and a 64-bp 13del-specific short probe (same as for the wt-specific probe, but with the 13-bp deleted) were generated. The probe for *BiP* corresponds to position 9–1,991 in mouse *BiP* mRNA. *BiP* cDNA was synthesized by RT-PCR and cloned into pBluescript II.

Marker analyses (with antibodies and/or riboprobes) were performed on sections from at least three 13del mice, and data shown is representative of consistent results.

### Generation of, and analyses using, *Col10a1-Cre* knock-in mice.

To express *Cre* recombinase specifically in HCs, a replacement gene-targeting vector was generated that contained *Col10a1* sequence extending from −2,070 to +7,680. This targeting vector was modified with *Cre* recombinase inserted at the ATG codon in exon 2 followed by frt-flanked neomycin resistance gene *(PGKneo),* such that the translated CRE proteins do not have a signal peptide and are not secreted. Gene targeting was carried out as described using R1 embryonic stem (ES) cells (gift of Andras Nagy) [67] Targeted ES clones were used to generate chimeras by blastocyst injection. Upon germline transmission of the *Col10a1-cre* gene, the PGKneo selection cassette was removed by crossing to β-actin-flp mice (gift of Susan Dymecki). To detect CRE expression and activity, *Col10a1-Cre* mice were bred to ROSA26 CRE reporter mice [68] and 13del mice. Tibiae of the compound mutant mice were collected for X-gal staining to detect β-galactosidase expression in mutant mice [68].

### Generation of EGFP/13del chimeras.

Homozygous albino (ICR) mice ubiquitously expressing EGFP were generated by crossing *β-actin Cre* transgenic mice (gift from Gail Martin) with the CRE reporter mouse, Z/EG [69]. EGFP/13del chimeras were created by aggregating morulae from the β-actin EGFP and 13del mice (CBA/C57BL6). The chimeras (white and agouti/black) generated were genotyped for the *Col10a1-13del* allele by PCR.

### Microscopy, image acquisition, and processing.

Fluorescence images were captured on an Axiovert 135 microscope (Carl Zeiss) using the Bio-Rad MRC-1024 laser scanning confocal imaging system (Bio-Rad, Hercules, California, United States). Pseudo-colored in situ hybridization images were created by overlaying bright field (blue filter) and dark field (red filter) images captured on a Axioplan 2 microscope (Carl Zeiss,) using a DKC-ST5 digital camera (Sony, Tokyo, Japan). The images were enhanced by adjusting the contrast with Photoshop 6.0 (Adobe Systems, San Jose, California, United States), and overlaid using the “Screen” blend mode of the “Layer” function to produce the final pseudo-colored image. For immunohistochemistry, images were captured on the same microscope with some images enhanced with the “Auto Levels” function in Photoshop 6.0 that automatically adjusts the tonal range to improve color contrast in each of the RGB channels with a default clip values of 0.5%.

## Supporting Information

Figure S1 Degree of HZ Expansion Correlated with Transgene Expression Level The relative transgene expression level for three independent 13del transgenic lines (13del, 13del-2, and 13del-3) was determined by RNase protection assay (see Materials and Methods for details), and the corresponding histology of proximal tibial growth plates shown. The transgene expression was normalized to 13del mice, the line on which we performed all our analyses. Three biological replicates were performed for each line and the relative expression indicated. The degree of HZ expansion (denoted by brackets) positively correlated with the relative transgene expression level. Bar indicates 100 μm.
(780 KB PDF)

Figure S2 Induction of *Edem* in 13del HCs In situ hybridization on tibial growth plates of new-born pups showing up-regulation of *Edem* expression (red) in 13del HZ. The insets show the dark field images of the same area. Bar indicates 100 μm.
(634 KB PDF)

Figure S3 13del Protein Expression Correlated with ERSS and Abnormal Terminal Differentiation (A) Co-localization of 13del and XBP1S in the same cells was observed in 18.5 dpc 13del UHZ of proximal tibia in which staining for 13del (brown) is cytoplasmic and that for XBP1S (blue) is nuclear.
(B) Co-localization of 13del and cyclin D1 in the same cells was observed in 10-d-old 13del LHZ of proximal tibia in which staining for 13del (brown) is cytoplasmic and that for cyclin D1 (blue) is nuclear. Staining for 13del was performed first, followed by heat treatment in boiling water to destroy the antibodies before the second staining for XBP1S or cyclin D1. Bar indicates 100 μm.
(869 KB PDF)

Figure S4 No Increase in Apoptosis Detected in 13del HCs TUNEL assay was performed on 10-d-old tibial paraffin sections using the In Situ Cell Death Detection Kit, Fluorescein (Roche), counterstained with propidium iodide to display the nuclei (red). Apoptotic cells with yellow nuclei (arrows) are present at the chondro-osseous junction in wt mice but not detected in 13del. Apoptotic cells are found in the bone marrow of wt and 13del mice (insets).
(3.3 MB PDF)

Figure S5 Reprogrammed Terminal Differentiation of 13del HCs Immunostaining and in situ hybridization of sections through the proximal tibial growth plate of 10-d-old mice.
(A) Immunostaining using SOX9 antibody (gift from Benoit de Crombrugghe) was performed at a dilution of 1:30 using Dako EnVision+ System as described [70]. In wt, note nuclear SOX9 in the PZ and pre-hypertrophic zone. In 13del, nuclear SOX9 is also found in cells in the LHZ: see higher magnification views of the boxed regions. In situ hybridization is shown for *Igf2* (B), *Col1a1* (C), and *Mmp13* (D).
(B) *Igf2* was expressed in wt RZ, PZ, and PH and down-regulated in HZ. In 13del, *Igf2* was re-expressed in the LHZ.
(C) In both wt and 13del mice, expression of *Col1a1* is restricted to bone with no expression in HCs. Higher magnifications of the boxed regions are shown to clearly demonstrate this differential expression.
(D) In wt mice, *Mmp13* is expressed in osteoblasts and terminally differentiated HCs. In 13del, expression is scattered in the LHZ.
Color contrast of (A) was adjusted as described in Materials and Methods. Bar indicates 100 μm.
(1.4 MB PDF)

Figure S6 Stress in the Growth Plate Chondrocytes of a Transgenic Mouse Expressing Mutant Collagen II Immunohistochemical detection of BiP in the proximal tibial growth plates of new-born mice homozygous for the *Col2a1G904C* transgene. Higher magnifications of the boxed regions are shown to demonstrate the elevated expression in *Col2a1G904C* transgenic mice in proliferating chondrocytes and disruption of the normal zonation and columnar structure of chondrocytes. Color contrast of the images was adjusted as described in Materials and Methods. The electron micrographs showed engorged ER in transgenic mice (arrows). Bar indicates 100 μm.
(1.8 MB PDF)

### Accession Numbers

The GenBank (http://www.ncbi.nlm.nih.gov/Genbank) accession numbers for the genes and gene products discussed in this paper are *Actinb* (NM_007393), *BiP* (NM_022310), *BiP* mRNA (AJ002387), *Col10a1* (NM_009925), *Mmp9* (NM_013599), and *Xbp1* mRNA (NM_013842).

## Abbreviations:

ATF6: activating transcription factor 6
BiP: binding Ig protein
bp: base pair
dpc: days post coitum
ECM: extracellular matrix
EGFP: enhanced green fluorescent protein
ER: endoplasmic reticulum
ERSS: ER stress signaling
HC: hypertrophic chondrocyte
HZ: hypertrophic zone
IHH: Indian hedgehog
IRE1: inositol-requiring 1
kb: kilobase
LHZ: lower hypertrophic zone
MCDS: metaphyseal chondrodysplasias type Schmid
PCNA: proliferating cell nuclear antigen
PERK: PKR-like ER kinase
PH: prehypertrophic zone
preHC: prehypertrophic chondrocyte
PTHrP: parathyroid hormone-related peptide
PZ: proliferating zone
UHZ: upper hypertrophic zone
wt: wild-type
*Xbp1^s^*: spliced variant of *Xbp1* mRNA
