# Supplementary material for: Surviving Endoplasmic Reticulum Stress Is Coupled to Altered Chondrocyte Differentiation and Function
Source: PLoS Biol. 2007 Feb 13;5(3):e44. doi: 10.1371/journal.pbio.0050044 (PMC1820825; doi:10.1371/journal.pbio.0050044)
Supplement: Figure S1 — The relative transgene expression level for three independent 13del transgenic lines (13del, 13del-2, and 13del-3) was determined by RNase protection assay (see Materials and Methods for details), and the corresponding histology of proximal tibial growth plates shown. The transgene expression was normalized to 13del mice, the line on which we performed all our analyses. Three biological replicates were performed for each line and the relative expression indicated. The degree of HZ expansion (denoted by brackets) positively correlated with the relative transgene expression level. Bar indicates 100 μm. (780 KB PDF) [file pbio.0050044.sg001.pdf]

Supplemental Fig. S1

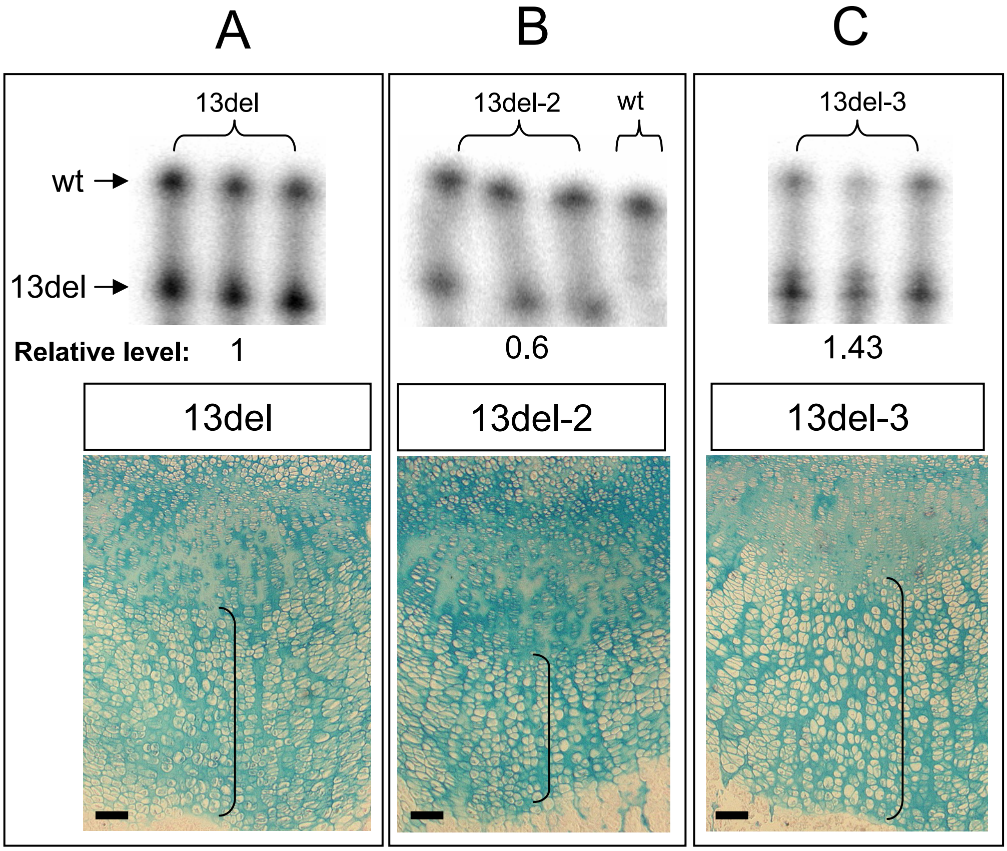

Figure S1. Degree of HZ expansion correlated with transgene expression level

The relative transgene expression level for three independent 13del transgenic lines (13del, 13del-2 and 13del-3) was determined by RNase protection assay (See “Materials and Methods” section for details), and the corresponding histology of proximal tibial growth plates shown. The transgene expression was normalized to 13del mice, the line which we performed all our analyses. Three biological replicates were performed for each line and the relative expression indicated. The degree of HZ expansion (denoted by brackets) positively correlated with the relative transgene expression level. Bar = 100µm.
