# Supplementary material for: Surviving Endoplasmic Reticulum Stress Is Coupled to Altered Chondrocyte Differentiation and Function
Source: PLoS Biol. 2007 Feb 13;5(3):e44. doi: 10.1371/journal.pbio.0050044 (PMC1820825; doi:10.1371/journal.pbio.0050044)
Supplement: Figure S2 — In situ hybridization on tibial growth plates of new-born pups showing up-regulation of Edem expression (red) in 13del HZ. The insets show the dark field images of the same area. Bar indicates 100 μm. (634 KB PDF) [file pbio.0050044.sg002.pdf]

Supplemental Fig. S2

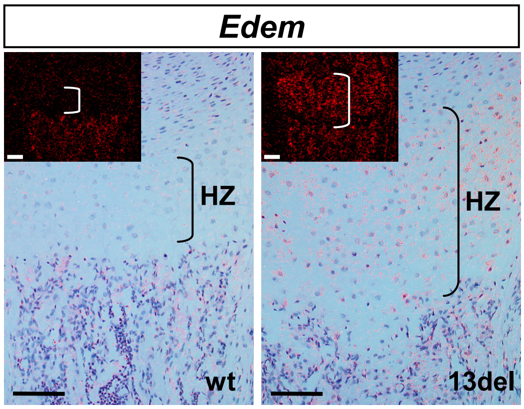

Figure S2. Induction of *Edem* in 13del hypertrophic chondrocytes

In situ hybridization on tibial growth plates of new born pups showing up-regulation of *Edem* expression (red) in 13del HZ. The insets show the dark field images of the same area. Bar = 100μm.
