# Supplementary material for: Surviving Endoplasmic Reticulum Stress Is Coupled to Altered Chondrocyte Differentiation and Function
Source: PLoS Biol. 2007 Feb 13;5(3):e44. doi: 10.1371/journal.pbio.0050044 (PMC1820825; doi:10.1371/journal.pbio.0050044)
Supplement: Figure S3 — (A) Co-localization of 13del and XBP1S in the same cells was observed in 18.5 dpc 13del UHZ of proximal tibia in which staining for 13del (brown) is cytoplasmic and that for XBP1S (blue) is nuclear. (B) Co-localization of 13del and cyclin D1 in the same cells was observed in 10-d-old 13del LHZ of proximal tibia in which staining for 13del (brown) is cytoplasmic and that for cyclin D1 (blue) is nuclear. Staining for 13del was performed first, followed by heat treatment in boiling water to destroy the antibodies before the second staining for XBP1S or cyclin D1. Bar indicates 100 μm. (869 KB PDF) [file pbio.0050044.sg003.pdf]

Supplemental Fig. S3

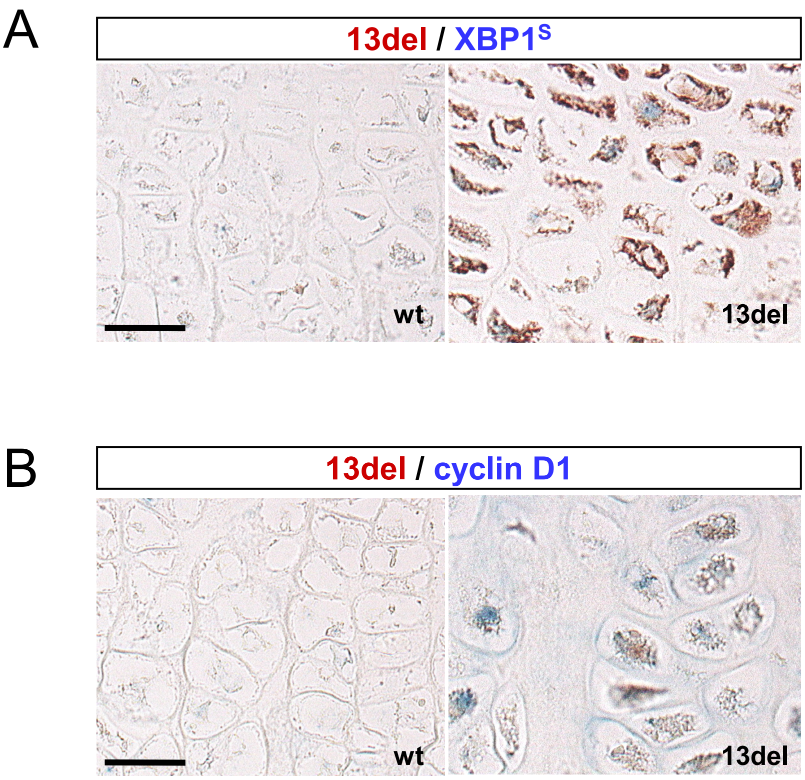

Figure S3. 13del protein expression correlated with ERSS and abnormal terminal differentiation

(A) Co-localization of 13del and $XBP1^S$ in the same cells was observed in 18.5 dpc 13del UHZ of proximal tibia where staining for 13del (brown) is cytoplasmic and that for $XBP1^S$ (blue) nuclear. (B) Co-localization of 13del and cyclin D1 in the same cells was observed in 10-day-old 13del LHZ of proximal tibia where staining for 13del (brown) is cytoplasmic and that for cyclin D1 (blue) nuclear. Staining for 13del was performed first followed by heat treatment in boiling water to destroy the antibodies before the second staining for $XBP1^S$ or cyclin D1. Bar = 100µm.
