# Supplementary material for: Surviving Endoplasmic Reticulum Stress Is Coupled to Altered Chondrocyte Differentiation and Function
Source: PLoS Biol. 2007 Feb 13;5(3):e44. doi: 10.1371/journal.pbio.0050044 (PMC1820825; doi:10.1371/journal.pbio.0050044)
Supplement: Figure S4 — TUNEL assay was performed on 10-d-old tibial paraffin sections using the In Situ Cell Death Detection Kit, Fluorescein (Roche), counterstained with propidium iodide to display the nuclei (red). Apoptotic cells with yellow nuclei (arrows) are present at the chondro-osseous junction in wt mice but not detected in 13del. Apoptotic cells are found in the bone marrow of wt and 13del mice (insets). (3.3 MB PDF) [file pbio.0050044.sg004.pdf]

Supplemental Fig. S4

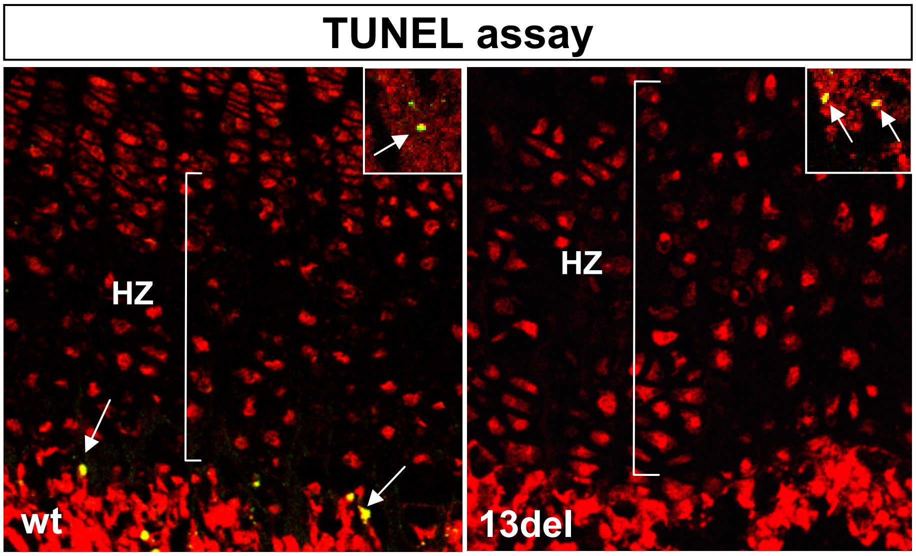

Figure S4. No Increase in Apoptosis Detected in 13del HCs

TUNEL assay was performed on 10-day-old tibial paraffin sections using the *In Situ* Cell Death Detection Kit, Fluorescein (Roche), counterstained with propidium iodide to display the nuclei (red). Apoptotic cells with yellow nuclei (arrows) are present at the chondro-osseous junction in wt but not detected in 13del. Apoptotic cells are found in the bone marrow of wt and 13del (insets). wt: wild type mice; 13del: 13del mice; HZ: hypertrophic zone.

Supplemental Fig. S5

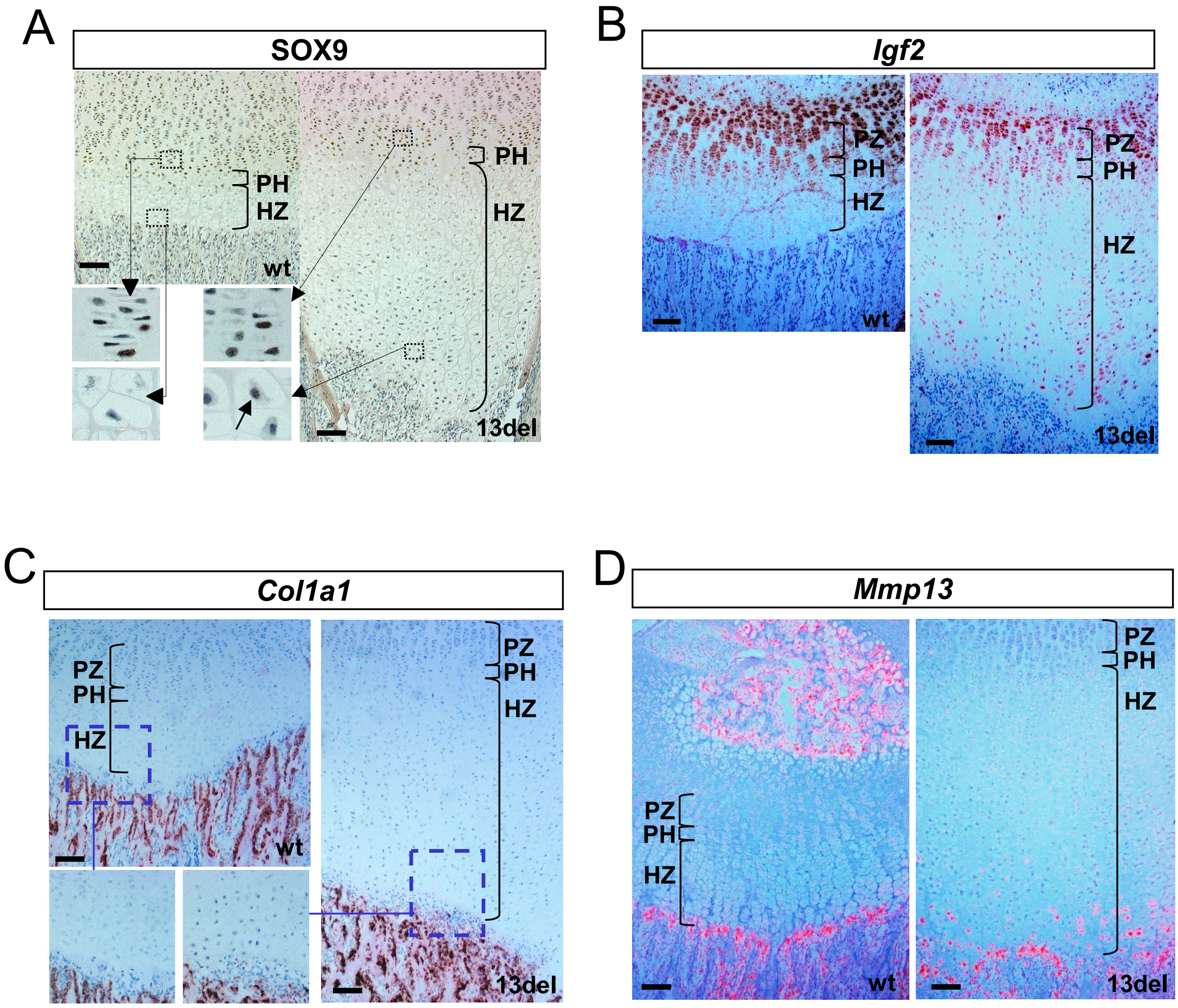

Figure S5. Reprogrammed terminal differentiation of 13del HCs

Immunostaining and *in-situ* hybridization of sections through the proximal tibial growth plate of 10-day-old mice. (A) Immunostaining using SOX9 antibody (gift from Benoit de Crombrugghe) was performed at a dilution of 1:30 using Dako EnVision+ System as described [Huang et al., (2000) *Mol. Cell Biol.* 20:4149]. In wt, note nuclear SOX9 in proliferating and pre-hypertrophic zones. In 13del, nuclear SOX9 is also found in cells in the LHZ: see higher magnification views of the boxed regions. *In-situ* hybridization for *Igf2* (B), *Col1a1* (C) and *Mmp13* (D). (B) *Igf2* was expressed in wt resting, proliferating and prehypertrophic zone and down-regulated in HZ. In 13del, *Igf2* was re-expressed in the LHZ. (C) In both wt and 13del, expression of *Col1a1* is restricted to bone with no expression in HCs. Higher magnifications of the boxed regions are shown to clearly demonstrate this differential expression. (D) In wt, *Mmp13* is expressed in osteoblasts and terminally differentiated HCs. In 13del, expression is scattered in the LHZ. wt: wild type mice; 13del: 13del mice; PZ: proliferating zone; PH: prehypertrophic zone; HZ: hypertrophic zone. Color contrast of panel A was adjusted as described in the “Materials and Methods” section. Bar = 100µm.

Supplemental Fig. S6

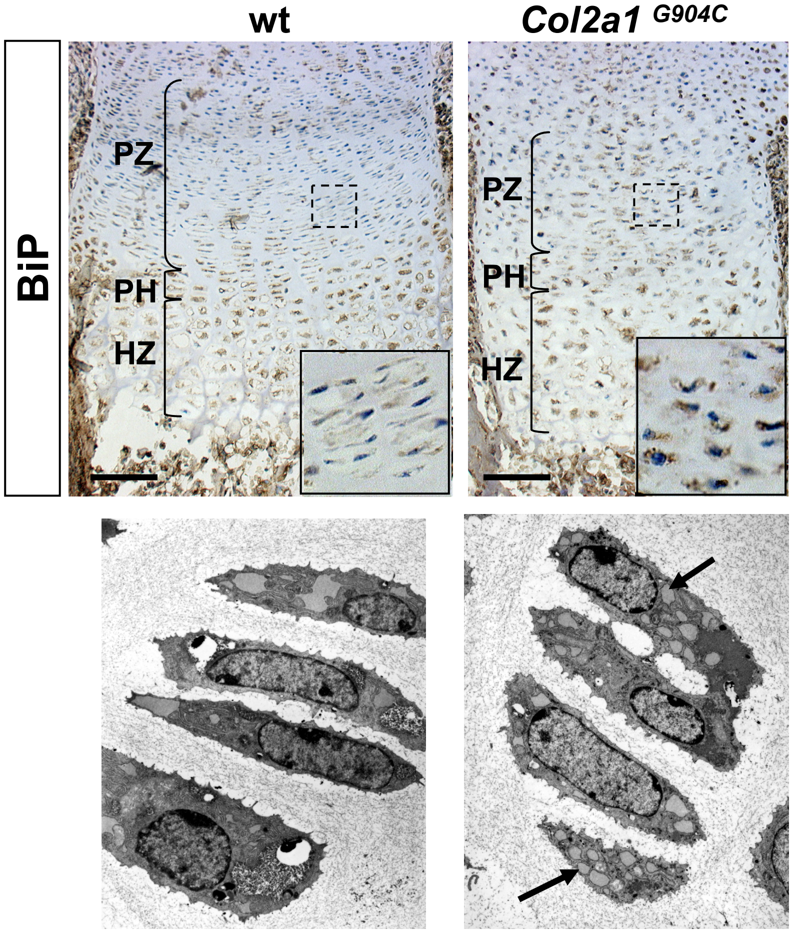

Figure S6. ER Stress in the Growth Plate Chondrocytes of a Transgenic Mouse Expressing Mutant Collagen II

Immunohistochemical detection of BiP in the proximal tibial growth plates of new born mice homozygous for the *Col2a1*^{G904C} transgene. Higher magnifications of the boxed regions are shown to demonstrate the elevated expression in *Col2a1*^{G904C} transgenic mice in proliferating chondrocytes and disruption of the normal zonation and columnar structure of chondrocytes. Color contrast of the images was adjusted as described in the "Materials and Methods" section. The electron micrographs showed engorged ER in transgenic mice (arrows). wt: wild type mice; 13del: 13del mice; PZ: proliferating zone; PH: prehypertrophic zone; HZ: hypertrophic zone. Bar = 100µm.
