# Supplementary material for: Surviving Endoplasmic Reticulum Stress Is Coupled to Altered Chondrocyte Differentiation and Function
Source: PLoS Biol. 2007 Feb 13;5(3):e44. doi: 10.1371/journal.pbio.0050044 (PMC1820825; doi:10.1371/journal.pbio.0050044)
Supplement: Figure S5 — Immunostaining and in situ hybridization of sections through the proximal tibial growth plate of 10-d-old mice. (A) Immunostaining using SOX9 antibody (gift from Benoit de Crombrugghe) was performed at a dilution of 1:30 using Dako EnVision+ System as described [70]. In wt, note nuclear SOX9 in the PZ and pre-hypertrophic zone. In 13del, nuclear SOX9 is also found in cells in the LHZ: see higher magnification views of the boxed regions. In situ hybridization is shown for Igf2 (B), Col1a1 (C), and Mmp13 (D). (B) Igf2 was expressed in wt RZ, PZ, and PH and down-regulated in HZ. In 13del, Igf2 was re-expressed in the LHZ. (C) In both wt and 13del mice, expression of Col1a1 is restricted to bone with no expression in HCs. Higher magnifications of the boxed regions are shown to clearly demonstrate this differential expression. (D) In wt mice, Mmp13 is expressed in osteoblasts and terminally differentiated HCs. In 13del, expression is scattered in the LHZ. Color contrast of (A) was adjusted as described in Materials and Methods. Bar indicates 100 μm. (1.4 MB PDF) [file pbio.0050044.sg005.pdf]

Supplemental Fig. S5

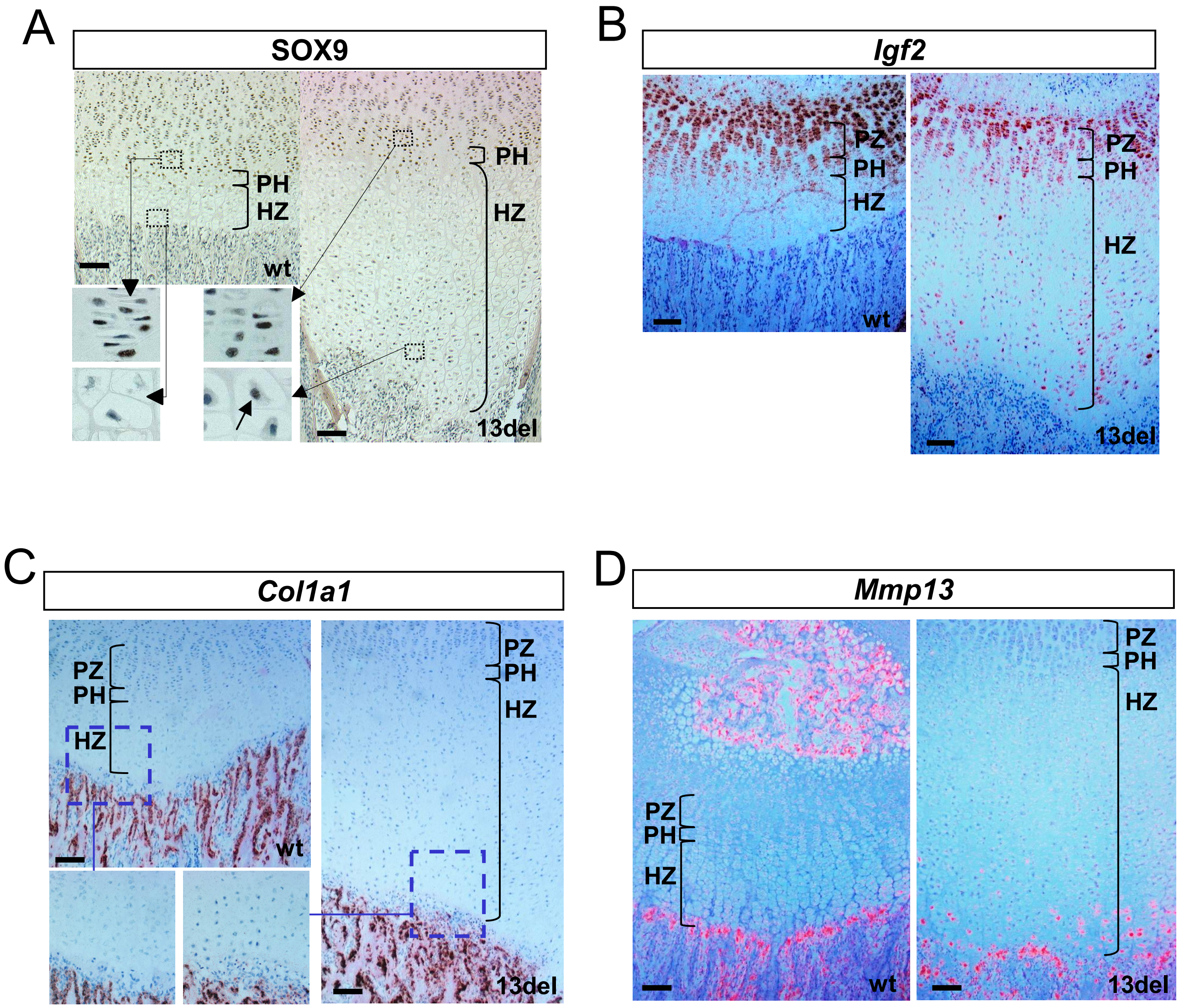

Figure S5. Reprogrammed terminal differentiation of 13del HCs

Immunostaining and *in-situ* hybridization of sections through the proximal tibial growth plate of 10-day-old mice. (A) Immunostaining using SOX9 antibody (gift from Benoit de Crombrugghe) was performed at a dilution of 1:30 using Dako EnVision+ System as described [Huang et al., (2000) *Mol. Cell Biol.* 20:4149]. In wt, note nuclear SOX9 in proliferating and pre-hypertrophic zones. In 13del, nuclear SOX9 is also found in cells in the LHZ: see higher magnification views of the boxed regions. *In-situ* hybridization for *Igf2* (B), *Col1a1* (C) and *Mmp13* (D). (B) *Igf2* was expressed in wt resting, proliferating and prehypertrophic zone and down-regulated in HZ. In 13del, *Igf2* was re-expressed in the LHZ. (C) In both wt and 13del, expression of *Col1a1* is restricted to bone with no expression in HCs. Higher magnifications of the boxed regions are shown to clearly demonstrate this differential expression. (D) In wt, *Mmp13* is expressed in osteoblasts and terminally differentiated HCs. In 13del, expression is scattered in the LHZ. wt: wild type mice; 13del: 13del mice; PZ: proliferating zone; PH: prehypertrophic zone; HZ: hypertrophic zone. Color contrast of panel A was adjusted as described in the “Materials and Methods” section. Bar = 100µm.
