# Supplementary material for: Surviving Endoplasmic Reticulum Stress Is Coupled to Altered Chondrocyte Differentiation and Function
Source: PLoS Biol. 2007 Feb 13;5(3):e44. doi: 10.1371/journal.pbio.0050044 (PMC1820825; doi:10.1371/journal.pbio.0050044)
Supplement: Figure S6 — Immunohistochemical detection of BiP in the proximal tibial growth plates of new-born mice homozygous for the Col2a1G904C transgene. Higher magnifications of the boxed regions are shown to demonstrate the elevated expression in Col2a1G904C transgenic mice in proliferating chondrocytes and disruption of the normal zonation and columnar structure of chondrocytes. Color contrast of the images was adjusted as described in Materials and Methods. The electron micrographs showed engorged ER in transgenic mice (arrows). Bar indicates 100 μm. (1.8 MB PDF) [file pbio.0050044.sg006.pdf]

Supplemental Fig. S6

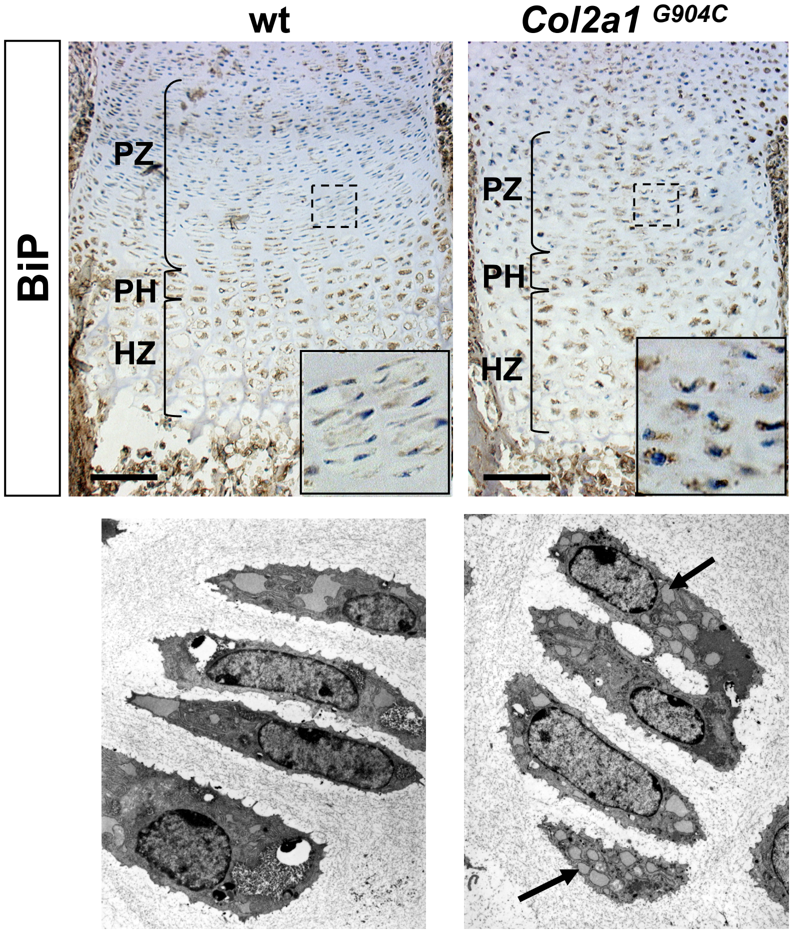

Figure S6. ER Stress in the Growth Plate Chondrocytes of a Transgenic Mouse Expressing Mutant Collagen II

Immunohistochemical detection of BiP in the proximal tibial growth plates of new born mice homozygous for the $\textit{Col2a1}^{G904C}$ transgene. Higher magnifications of the boxed regions are shown to demonstrate the elevated expression in $\textit{Col2a1}^{G904C}$ transgenic mice in proliferating chondrocytes and disruption of the normal zonation and columnar structure of chondrocytes. Color contrast of the images was adjusted as described in the "Materials and Methods" section. The electron micrographs showed engorged ER in transgenic mice (arrows). wt: wild type mice; 13del: 13del mice; PZ: proliferating zone; PH: prehypertrophic zone; HZ: hypertrophic zone. Bar = 100µm.
